# Deciphering Adaptation Strategies of the Epidemic *Clostridium difficile* 027 Strain during Infection through *In Vivo* Transcriptional Analysis

**DOI:** 10.1371/journal.pone.0158204

**Published:** 2016-06-28

**Authors:** Imad Kansau, Amira Barketi-Klai, Marc Monot, Sandra Hoys, Bruno Dupuy, Claire Janoir, Anne Collignon

**Affiliations:** 1 EA4043 Unité Bactéries Pathogènes et Santé (UBaPS), Univ. Paris-Sud, Université Paris-Saclay, 92296, Châtenay-Malabry Cedex, France; 2 Laboratoire Pathogenèse des Bactéries Anaérobies, Institut Pasteur, 25–28, rue du Docteur Roux, 75015, Paris, France; Cornell University, UNITED STATES

## Abstract

*Clostridium difficile* is responsible for a wide spectrum of infection from asymptomatic carriage to severe, relapsing colitis. Since 2003, *C*. *difficile* infections have increased with a higher morbidity and mortality due to the emergence of epidemic and hypervirulent *C*. *difficile* strains such as those of the epidemic lineage 027/BI/NAP1. To decipher the hypervirulence and epidemicity of 027 strains, we analyzed gene expression profiles of the R20291 027 strain using a monoxenic mouse model during the first 38h of infection. A total of 741 genes were differentially expressed during the course of infection. They are mainly distributed in functional categories involved in host adaptation. Several genes of PTS and ABC transporters were significantly regulated during the infection, underlying the ability of strain R20291 to adapt its metabolism according to nutrient availability in the digestive tract. In this animal model, despite the early sporulation process, sporulation efficiency seems to indicate that growth of R20291 vegetative cells versus spores were favored during infection. The bacterial mechanisms associated to adaptability and flexibility within the gut environment, in addition to the virulence factor expression and antibiotic resistance, should contribute to the epidemicity and hypervirulence of the *C*. *difficile* 027 strains.

## Introduction

*Clostridium difficile* is a Gram positive, spore forming, anaerobic bacterium and is the major cause of nosocomial intestinal disease associated with antibiotic therapy. *C*. *difficile* infection (CDI) has reached an epidemic state with increasing incidence and severity both in healthcare and community settings. This rise in morbidity and mortality results from emergence of the *C*. *difficile* epidemic lineage 027/BI/NAP1, whose strains have spread throughout developed countries [1,2]. The spectrum of infection, ranging from asymptomatic carriage to fulminant relapsing colitis, depends on *C*. *difficile* virulence factors, the fitness of the bacteria within the gastrointestinal tract and host susceptibility.

The pathogenesis of CDI begins by the disruption of the normal colonic microbiota by antibiotics allowing the germination of contaminating spores and the colonization of the gastrointestinal tract by vegetative forms [3]. Several *C*. *difficile* surface proteins potentially function as colonization factors. Thus it has been shown that the two S-layer subunits [4], the Cwp66 protein [5], the fibronectin-binding protein Fbp68 [6], the collagen-binding protein CbpA [7] and the lipoprotein CD0873 [8] all play a role in the adherence of bacteria to the epithelial cells. In addition, flagellar proteins also display adhesive properties [9] while proteases such as Cwp84 or Zmp1 may participate in the dissemination of the bacteria [10–12]. The last step corresponds to the production of the major virulence factors of *C*. *difficile*, the toxins TcdA and TcdB that modify the actin skeleton of the intestinal cells by glucosylation of the Rho proteins [13–16]. A third toxin, the binary toxin is produced by some strains such as the 027 strains, which possibly potentiates toxicity of TcdA and TcdB, and leads to more severe diseases [17].

The epidemic features of the 027 strains might be explained by their antibiotic resistance profile and sporulation rates [18,19], while sporulation characteristics vary according to experimental conditions and isolates [20]. In addition, the 027 strains exhibit a higher production of toxins [19,21,22], although the mechanism is not clearly understood. Nevertheless, the hypervirulence of these strains is still not fully understood.

In order to improve our knowledge of the hypervirulence mechanisms of the 027 strains, we analyzed the genome-wide temporal expression of the 027 strain R20291 using a monoxenic mouse model during the first 38h of infection. This animal model was previously successfully applied to decipher some of the adaptation mechanisms of *C*. *difficile* strain 630 in the host [12]. Here, we analyzed the kinetics of gene expression of strain R20291 at 4, 6, 8, 14 and 38h post infection. We found that 741 genes were regulated during *in vivo* growth. The modulation of expression mainly concerned transport, metabolism and sporulation genes. This work led to new insights into adaptation strategies used by the 027 strains during the infection process.

## Material and Methods

### Bacterial strains and growth conditions

Strain R20291 (Wild-type BI/NAP1/027 Stoke Mandeville isolate, gift from N. Minton, University of Notthingham, UK) was cultured in peptone yeast (PY) agar or broth (Oxoid) in an anaerobic atmosphere (10% CO_2_, 10% H_2_, 80% N_2_) at 37°C. Sporulation assays were performed from cell cultures grown in BHIS (brain heart infusion containing 5 mg ml^-1^ yeast extract [AES] and 0.1% [w/v] L-cysteine [Merck]). After heat shock treatment (60°C, 30 min), spores were quantified (CFU/ml) on BHIS agar supplemented with 0.1% sodium taurocholate to induce germination.

### Gnotobiotic mouse model

Animal care and experiments were carried out in accordance with the Committee for Research and Ethical Issues of the International Association for the Study of Pain (IASP). The Animal Welfare Committee of the Paris Sud University approved the animal experimentation protocol. C3H/HeN germ-free 6–8 week old mice (CNRS, Orléans, France), housed in sterile isolators, received sterilized standard nutrients and water. Groups of 6 axenic mice were challenged by oral gavage with 1x10^8^ CFUs of R20291 vegetative cells (spore rate less than 0.1% of vegetative cells) from early stationary phase [23]. After challenge with *C*. *difficile*, the monoxenic-associated mice did not exhibit symptoms indicative of severe illness and survived the entire experiment. Mice were humanely euthanized according to the guidelines of the "The Animal Welfare Committee of the Paris Sud University" at 4h, 6h, 8h, 14h and 38h, and cecal contents were collected. Another group of 6 mice was used to measure *C*. *difficile* fecal shedding from 4h to 148h post-infection by plating fecal dilutions either on BHI (for vegetative cell enumeration) or after a heat shock treatment (60°C, 30 min) on BHI supplemented with 0.1% of sodium taurocholate (for spore enumeration). The toxin production was measured in the cecal contents of mice qualitatively with the "Ridascreen^R^ Clostridium difficile ToxinA/B (r-biopharm) and quantitatively with a cell cytotoxicity assay.

### RNA extraction

Bacterial RNA was extracted from the cecal contents recovered at the different times, as previously described [12]. Both RNA quality and quantity were analyzed on the Bionalyser Agilent 2100 and RNA 6000 Nano Reagents (Agilent) and by qPCR on housekeeping genes *gyrA* and *rpoA*. According to the amount of RNA extracted and its quality (RINs>7), the four best samples per time point were used for transcriptomic analysis.

### Reverse transcription and microarray hybridizations

The microarray of *C*. *difficile* R20291 genome (GEO database accession number GPL15218) was designed as previously described [23]. Competitive hybridization assays and data analysis were performed as previously described [23]. A gene was considered as differentially expressed when the p-value was <0.05. The complete data set was deposited in the GEO database with a series record accession number GSE35726: http://www.ncbi.nlm.nih.gov/geo/query/acc.cgi?acc=GSE35726.

### Real-time reverse transcription PCR (qRT-PCR) analysis of gene expression

Quantitative RT-PCR was performed as indicated previously [23] to confirm the regulation of 14 selected genes. The results were normalized using the geometric averaging of 4 reference genes (*polII*, *rrs*, *rpoA* and *gyrA*). Normalized relative quantities were calculated using the ΔΔCT method. The Mann-Whitney test was performed using StatEL software to determine statistical significant difference (p < 0.05).

## Results

### Global analysis of strain R20291 gene expression during *in vivo* growth

**Fig 1:**
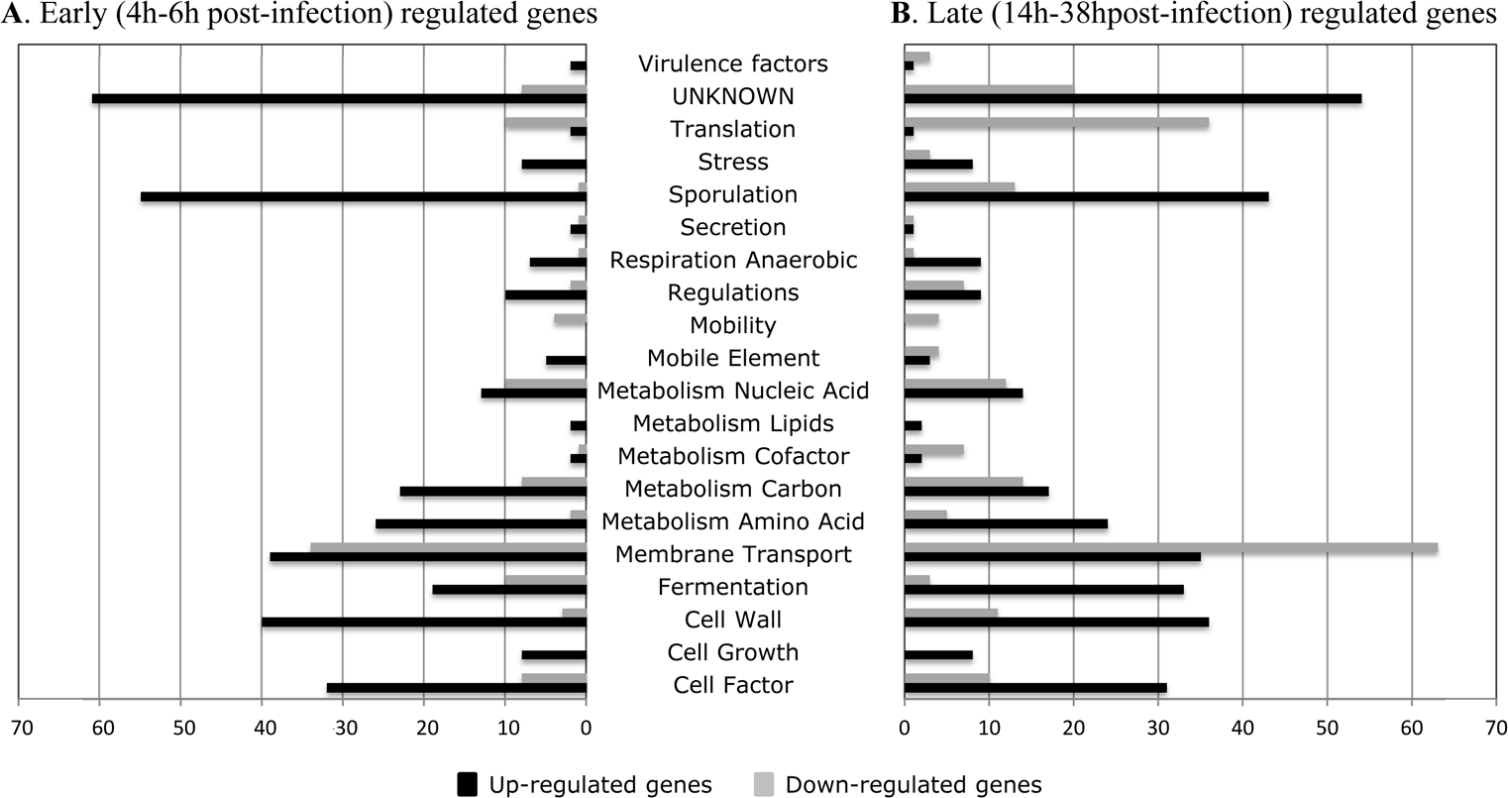
Functional clusters of in vivo differentially expressed genes (p-value < 0.05) in the *C*. *difficile* strain R20291. Genes were classified in functional groups according to functions. Black and grey bars represent respectively up-regulated and down-regulated genes. (A) Regulated genes at 4 and 6h compared to 8h. (B) Regulated genes at 14 and 38h compared to 8h.

**Fig 2:**
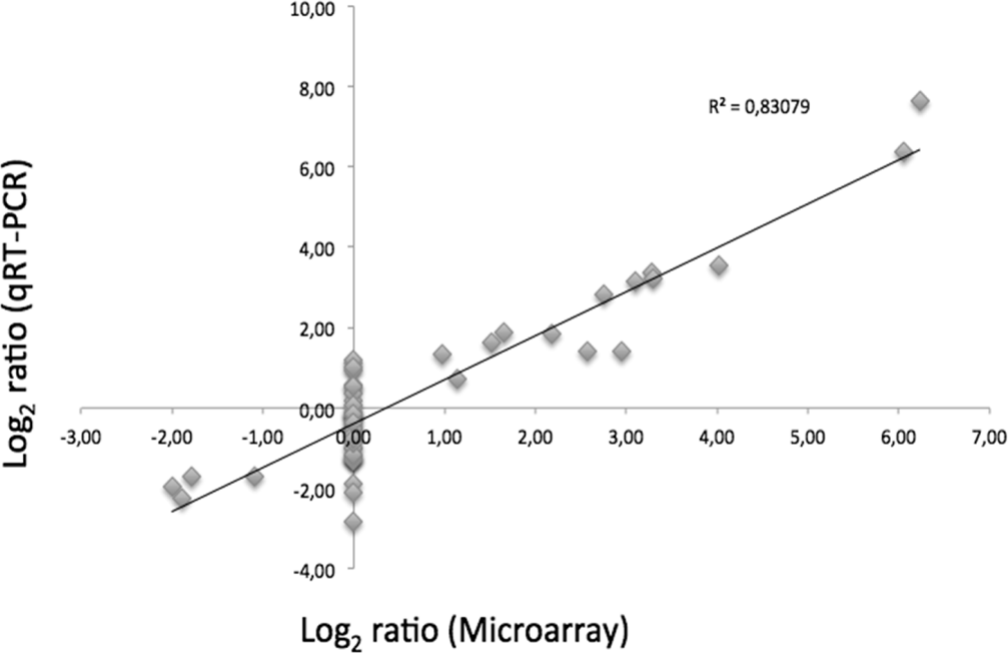
Validation of microarray data by qRT-PCR. Fold changes in in vivo gene expression at 4, 6, 14 and 38h post-infection, compared to the in vivo expression at 8h post-infection, were measured by microarray and qRT-PCR. Data are plotted as log_2_ ratios of microarrays data (*x*-axis) compared with those of qRT-PCR (*y*-axis).

A total of 741 genes exhibited differential expression during the course of mouse infection. Among these genes, 285 genes were upregulated (fold change ≥ 2) while 456 were downregulated (fold change ≤ 0.5) at early (4-6h) and/or late (14-38h) infection time relative to 8h post-challenge (Table A in S1 File). All genes found differentially expressed were then assigned to functional categories. The classification was done manually on differentially expressed operons using the functional categories defined previously [12] (Fig 1). The results of the qRT-PCR experiments performed on 14 selected genes confirmed the trends observed by the microarray data with a high correlation coefficient (R = 0.83) (Fig 2, Table B in S1 File) and validated the transcriptomic profile data. All biological processes whose constituent genes were either up- or down-regulated could have a possible role in survival in vivo, and thus were given special attention.

### Expression of genes encoding virulence factors

Transcriptional profiles of toxin genes only showed a significant upregulation of *tcdA* and *tcdB* at 38h post-infection. In addition, *tcdE* was upregulated simultaneously at 38h, which supports the late release of toxins during growth [24]. This is consistent with *in vitro* results showing that toxin genes are expressed at a late stage of growth in response to the environmental status [25]. In our experiments, toxins were positive in the cecal samples with a cytotoxicity titer 1:40000 at 48h post-infection. Interestingly, genes encoding the binary toxin were induced early (8h) and were constitutively expressed thereafter (Table 1 and Table B in S1 File).

**Table 1:** Regulation of toxin genes during the kinetics of infection.

Gene ID CDR20291	Gene ID CD630	Gene name	Gene product description	Fold change compared to 8h				
				4h	6h	8h	14h	38h
CDR0581	CD0659	*tcdR*	Alternative RNA polymerase sigma factors	1.00	1.00	1.00	1.00	1.00
CDR0582	CD0660	*tcdB*	Toxin B	1.00	1.00	1.00	1.00	**4.53**
CDR0583	CD0661	*tcdE*	Holin-like pore-forming protein	1.00	1.00	1.00	1.00	**1.96**
CDR0584	CD0663	*tcdA*	Toxin A	**0.47**	1.00	1.00	1.00	**2.87**
CDR0585	CD0664	*tcdC*	Negative regulator of toxin gene expression	1.00	1.00	1.00	1.00	1.00
CDR2490		*cdtR*	Binary toxin regulatory gene LytTR family	1.00	1.00	1.00	1.00	1.00
CDR2491		*cdtA*	Fragment of ADP-ribosyltransferase	1.00	1.00	1.00	1.00	1.00
CDR2492		*cdtB*	Fragment of ADP-ribosyltransferase	**0.27**	**0.29**	1.00	1.00	1.00

Genes upregulated or downregulated by a fold change of 1.5 or more are in bold

Several genes encoding surface proteins and known colonization factors such as *slpA*, *cwp66*, *cwp84*, *fbpA* and *cpbA* were either not expressed or not differentially regulated during the course of infection. However, the CDR0802 (CD630_08730) gene, encoding a surface protein involved in the adhesion process [8], was highly up- and down-regulated over time, with a gradual upregulation from 6h to 14h, followed by a downregulation at 38h post-challenge (Table C in S1 File). In addition, some genes encoding surface proteins with unknown or putative function were differentially regulated during the growth such as *cwp25* (CDR0774) and *cwp10* (CDR2685) upregulated early and late, respectively, and the gene coding for a putative fibronectin-binding protein (CDR2686), upregulated at 4h and 38h post-infection (Table C in S1 File). Surprisingly, flagellar and type IV pilus encoding genes were not regulated during *in vivo* infection.

### Expression of transport and metabolism encoding genes

Many genes involved in metabolic functions were upregulated during mouse infection and especially those involved in membrane transports and carbohydrate, lipid and amino acid metabolism (Tables A, D and E in S1 File)**.** Most of the PTS genes of strain R20291 were not regulated *in vivo*. However, we found that genes coding for two PTS systems of the lactose/cellobiose family (CDR0133-0137, CDR3263-3267) were upregulated early (4h and 6h, respectively) while others were expressed late. Among the latter, we found that genes encoding two PTS glucose-like (CDR2401-2404, CDR2862-2866) and one PTS mannose/fructose operon (CDR3136-3140) were upregulated at 14-38h and 38h, respectively. Interestingly, the *gatABC* genes (CDR2214-2216) of the PTS galactitol and *gatD* (CDR2213) encoding a galactitol dehydrogenase, were highly upregulated early and late during infection. Galactitol is produced by reducing galactose and is then converted by *gatD* to D-tagatose-6-phosphate. Interestingly, we noted that a PTS tagatose system (CDR2913-2915) was also upregulated during early colonization (Table D in S1 File). In *Enterobacteriaceae*, these two pathways generate glyceraldehyde-phosphate (GAP) and dihydroxyacetone-phosphate (DHAP) that could be used through the glycolysis pathway to generate pyruvate and ATP [26]. Thus, according to their availability in the gut, strain R20291 may have a preferred use of carbohydrate sources to produce pyruvate during the course of the infection.

**Fig 3:**
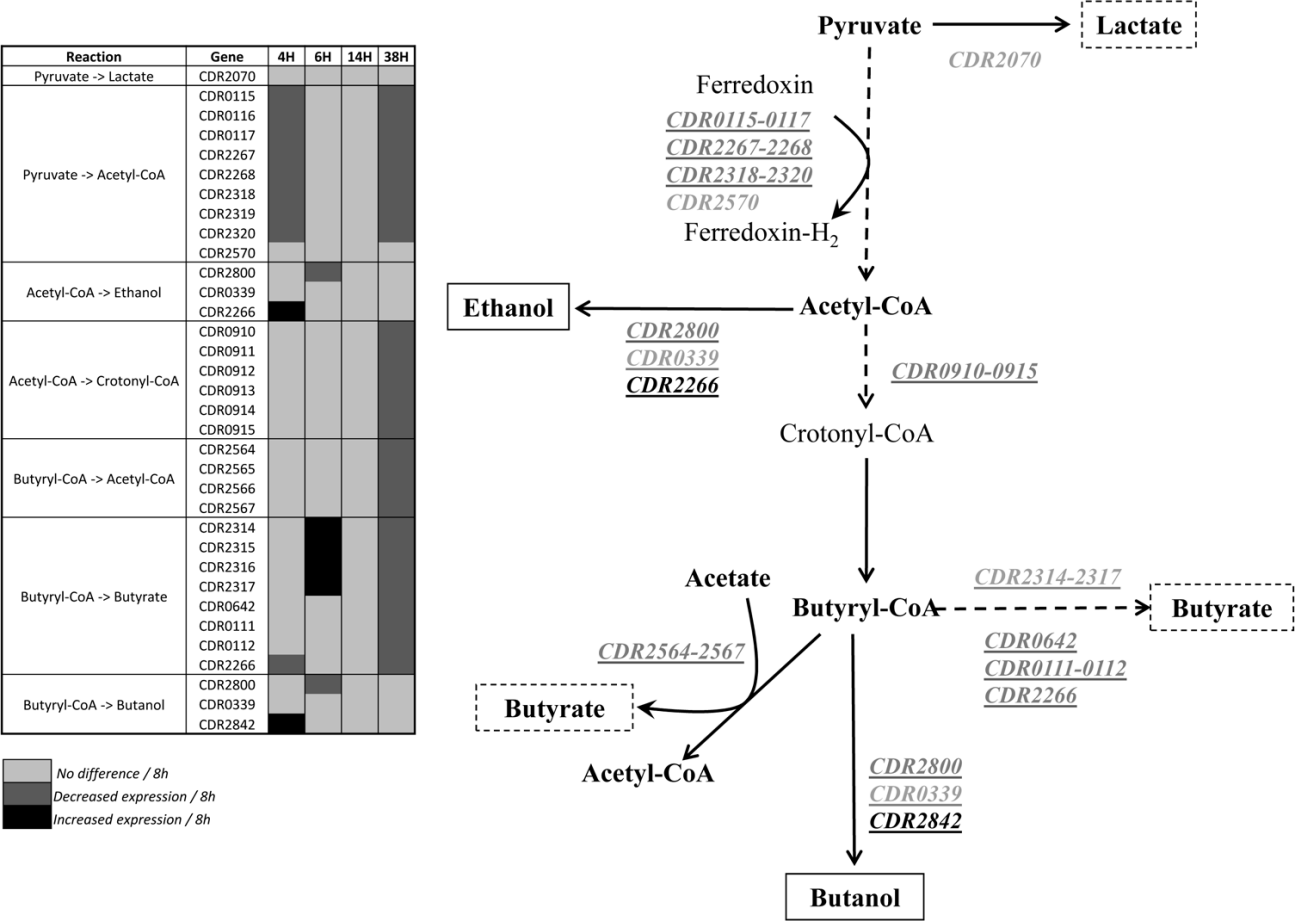
Gene regulation in the fermentation pathway involved in the production of butyrate, butanol and ethanol.

Pyruvate is then metabolized by various fermentation pathways for energy production or used for anabolic reactions. We found that genes encoding enzymes involved in the synthesis of acetyl-coenzyme A (acetyl-CoA), i.e. indolepyruvate ferredoxin oxidoreductase (CDR2267-2268) and the putative ferredoxin/flavodoxin oxidoreductases (CDR0115-0117 and CDR2318-2320), were downregulated at 4h and 38h (Table D in S1 File), suggesting that they were expressed at the mid-phase (6-14h) of the colonization. In addition, we observed that genes encoding enzymes involved in the conversion of acetyl-CoA into butyryl-CoA via an intermediary of crotonyl-CoA (CDR0910-0915) were constitutively expressed since they are downregulated at 38h. Moreover, several gene clusters encoding enzymatic pathways involved in the final synthesis of butyrate from butyryl-CoA (CDR2314-2317; CDR2565-2567) or acetate (CDR2564-2567) were differentially regulated during the course of infection. However, they all showed a downregulation at 38h (Fig 3, Table D in S1 File), suggesting a coordinate decreased production of butyrate at 38h. Among the other genes differentially regulated *in vivo* and associated with the carbohydrate metabolism, we found that genes encoding the ribose-5-phosphate isomerase (CDR2209) and the transketolase (CDR2210-2211) involved in the pentose phosphate pathway and in connection with the glycolytic pathway, respectively, were upregulated early (4-6h) and late (38h) during infection (Table D in S1 File).

Several ABC transporters were upregulated late during the course of infection (Tables A and E in S1 File). Of particularl interest, operons *appABC* and *appDF* encoding oligopeptide transporters (CDR2558-2562) and genes encoding cystein transporter (CDR2080-2083) or multidrug transporter (CDR1501-1503) were upregulated at 14h and 38h. Peptide and amino-acid assimilation by the cell can be used as carbon and nitrogen sources and metabolized through the Stickland reaction to produce ATP and to regenerate the redox status [25]. In contrast, the operons *opuCA* and *opuCC* encoding Glycine betaine ABC transporter (CDR0830-0831) were first upregulated early (6h), but were then downregulated at 38h. Glycine betaine is an excellent osmoprotectant and is widely used by prokaryotes as an adaptive strategy to high osmolarity environment, such as gut content [27].

### Stress response genes

During infection, *C*. *difficile* encounters several environment stresses and must adapt to them. This requires a wide range of mechanisms, involving tightly regulated and coordinated protein expression. The more striking result was the late 38h upregulation of several genes encoding class I Heat Shock Proteins (HSP), such as *dnaJ*, *dnaK* and *gprE* (CDR2353-2355), and the regulatory gene *hcrA* (CDR2356) (Table A in S1 File). This is consistent with a comparative proteomic study performed by Chen et al. [28], that shows that epidemic 027 strain R20291 has a more robust stress response than the historic non-epidemic 027 strain (CD196), which might explain why the epidemic 027 strains adapt better during colonization.

### Sporulation genes

Several genes involved in different stages of the sporulation process were regulated during mice infection (Table 2). Among them, we found that expression of *spo0A* gene coding for the main transcriptional regulator of sporulation [29] was upregulated early (6h) before being downregulated at 38h, suggesting that *in vivo* the sporulation process begins early for strain R20291. Accordingly, some genes known to be dependent on Spo0A, such as *spoIIA* operon coding for the sporulation sigma factor σ^F^ (CDR0701) and proteins that control its activity in the forespore (CDR0699 and CDR0700), were regulated similarly (Table 2). The other sporulation-specific sigma factors σ^E^ (CDR2531) and σ^G^ (CDR2530), controlling gene expression in the mother cell and in the forespore, respectively, were mainly expressed à 8h post-infection (Table 2). In agreement, expression of the σ^E^-dependent *spoIIIA* operon (CDR1030-CDR1037), encoding an ATPase and membrane proteins localized in the outer membrane of the forespore was also similarly upregulated at 8h post-infection, as well as some σ^G^ dependent genes like *sspA* (CDR2576) and *sspB* (CDR3107), encoding small acid-soluble proteins (SASP) which are responsible for the protection of the forespore chromosome. Late upregulated genes mainly belong to the *sigK* regulon [30,31]. This concerns genes encoding dipicolinate synthase subunits (CDR2802-2803) and spore surface proteins encoding genes (Table 2). For instance, *cotJA*, *cotJB1* encoding spore coat proteins and *cotCB* encoding a putative manganese catalase present in the coat were highly upregulated. Similarly, expression of *bclA* genes encoding exosporium glycoproteins and *cdeC* encoding a cysteine-rich exosporium protein were upregulated late during infection (Table 2).

**Table 2:** Regulation of sporulation and germination genes during the kinetics of infection.

Gene ID CDR20291	Gene ID CD630	Gene Name	Gene product description	Fold change compared to 8h				
				4h	6h	8h	14h	38h	
CDR0123	CD0124	*spoIIC*	Stage II sporulation protein C	**0.42**	**0.24**	1.00	1.00	**0.45**	
CDR0125	CD0126	*spoIIID*	Stage III sporulation protein D	**0.11**	**0.12**	1.00	**0.32**	**0.15**	
CDR0521	CD0596	*cotJA*	Hypothetical protein	1.00	1.00	1.00	**72.50**	**54.30**	
CDR0522	CD0597	*cotF*	Spore coat protein	1.00	1.00	1.00	**74.91**	**66.46**	
CDR0523	CD0598	*cotCB*	Spore coat protein CotCB manganese catalase	1.00	1.00	1.00	**66.46**	**59.32**	
CDR0699	CD0770	*spoIIAA*	Anti-sigma F factor antagonist	**0.08**	1.00	1.00	1.00	**0.26**	
CDR0700	CD0771	*spoIIAB*	Anti-sigma F factor	**0.10**	1.00	1.00	1.00	**0.28**	
CDR0701	CD0772	*sigF*	RNA polymerase sigma-F factor	**0.20**	1.00	1.00	1.00	**0.31**	
CDR0702	CD0773	*spoVAC*	stage V sporulation protein AC	**0.24**	**0.26**	1.00	1.00	1.00	
CDR0703	CD0774	*spoVAD*	stage V sporulation protein AD	**0.31**	**0.29**	1.00	1.00	**0.33**	
CDR0704	CD0775	*spoVAE*	stage V sporulation protein AE	**0.39**	**0.43**	1.00	1.00	1.00	
CDR0714	CD0783	*spoIVB*	Stage IV sporulation protein SpoIVB, 55 peptidase family	1.00	1.00	1.00	1.00	**3.05**	
CDR0926	CD1067	*cdeC*	Cysteine-rich exosporium protein	**0.10**	**0.08**	1.00	**10.15**	**7.42**	
CDR1030	CD1192	*spoIIIAA*	Stage III sporulation protein AA	**0.14**	**0.26**	1.00	0.34	0.15	
CDR1031	CD1193	*spoIIIAB*	Stage III sporulation protein AB	**0.22**	**0.20**	1.00	0.30	0.28	
CDR1032	CD1194	*spoIIIAC*	Stage III sporulation protein AC	**0.20**	**0.20**	1.00	**0.26**	**0.22**	
CDR1033	CD1195	*spoIIIAD*	Stage III sporulation protein AD	**0.18**	**0.18**	1.00	**0.24**	**0.17**	
CDR1034	CD1196	*spoIIIAE*	Stage III sporulation protein AE	**0.23**	**0.24**	1.00	**0.27**	**0.22**	
CDR1035	CD1197	*spoIIIAF*	Stage III sporulation protein AF	**0.30**	**0.27**	1.00	**0.34**	**0.34**	
CDR1036	CD1198	*spoIIIAG*	Stage sporulation protein AG	**0.05**	**0.06**	1.00	**0.26**	**0.09**	
CDR1037	CD1199	*spoIIIAH*	Stage III sporulation protein AH	**0.06**	**0.07**	1.00	**0.26**	**0.10**	
CDR1052	CD1214	*spo0A*	Stage 0 sporulation protein A	**0.25**	1.00	1.00	1.00	**0.40**	
CDR1511	CD1613	*cotA*	Spore outer coat layer protein	1.00	1.00	1.00	**19.35**	**15.89**	
CDR1529	CD1631	*sodA*	Spore coat superoxyde dismutase	**0.26**	**0.27**	1.00	1.00	1.00	
CDR1858	CD1935	*spoVS*	stage V sporulation protein S	**0.20**	1.00	1.00	1.00	**0.34**	
CDR2290	CD2400	*cotJB2*	Spore coat protein JB 2	**0.16**	**0.15**	1.00	**5.98**	1.00	
CDR2291	CD2401	*cotD*	Putative manganese catalase	**0.16**	**0.16**	1.00	**6.89**	**3.91**	
CDR2334	CD2442	*spoIV*	Stage IV sporulation protein	**0.28**	**0.29**	1.00	1.00	**0.35**	
CDR2362	CD2469	*spoIIP*	Stage II sporulation protein P	**0.25**	**0.23**	1.00	**0.37**	**0.24**	
CDR2363	CD2470	*gpr*	Spore endodpeptidase	**0.22**	**0.26**	1.00	**0.36**	**0.21**	
CDR2513	CD2629	*spoIVA*	Stage IV sporulation protein A	**0.04**	**0.04**	1.00	**0.31**	**0.09**	
CDR2530	CD2642	sigG	Sporulation sigma factor G	**0.06**	**0.09**	1.00	**0.25**	**0.10**	
CDR2531	CD2643	*sigE*	Sporulation sigma factor E	**0.13**	**0.19**	1.00	**0.25**	**0.17**	
CDR2532	CD2644	*spoIIGA*	Sporulation sigma factor E processing peptidase	**0.29**	**0.34**	1.00	**0.34**	**0.29**	
CDR2576	CD2688	*sspA*	Small acid-soluble spore protein A	**0.02**	**0.01**	1.00	1.00	1.00	
CDR2802	CD2967	*spoVFB*	Dipicolinate synthase subunit B	**0.33**	**0.33**	1.00	**2.50**	1.00	
CDR2803	CD2968	*dpaA*	Dipicolinate synthase subunit B	**0.28**	**0.29**	1.00	**2.59**	1.00	
CDR3090	CD3230	*bclA2*	Exosporium glycoprotein	1.00	1.00	1.00	**5.96**	**3.15**	
CDR3107	CD2688	*sspB*	small acid-soluble spore protein B	**0.04**	**0.03**	1.00	1.00	1.00	
CDR3193	CD3349	*bclA3*	Putative exosporium glycoprotein	**0.24**	**0.26**	1.00	**5.69**	1.00	
CDR3327	CD3490	*spoIIE*	Stage II sporulation protein E	**0.01**	**0.09**	1.00	**0.36**	**0.09**	
CDR3401	CD3564	*spoIIR*	Stage II sporulation protein R	**0.35**	**0.31**	1.00	1.00	**0.36**	
CDR3404	CD3567	*sipL*	SpoIVA interacting protein	**0.05**	**0.05**	1.00	**0.36**	**0.10**	
CDR3336	CD3499	*spoVT*	stage V sporulation protein S	**0.09**	**0.09**	1.00	**0.34**	**0.14**	
CDR3353	CD3516	*spoVG*	stage V sporulation protein G	**0.22**	1.00	1.00	1.00	**0.44**	

Genes upregulated or downregulated by a fold change of 1.5 or more are in bold

### Specific R20291 genes

The 027 genomes have 234 additional genes compared to the 630 genome [32,33], including genes of a 20 kb phage island, and several transcriptional regulators, among them, a complete copy of an *agr* system named *agr2*. Our transcriptomic analysis did not show clear modulation of specific 027 gene expression during the course of infection. In particular, neither genes belonging to the phage island nor genes coding for the Agr2 system, were regulated in vivo. However, we cannot exclude that they are steadily expressed over time since they have been shown to contribute to the fitness and the virulence potential of the *C*. *difficile* 027 strains [34]. Finally, the *thyA* gene and the associated genes (CDR0044, 0045, 0047) were upregulated early (4h) and constitutively expressed thereafter (Table A in S1 File). The *thyX* gene present in strain 630, is replaced in strain R20291 by the *thyA* gene coding for a thymidylate synthase involved in the synthesis of dTMP with a much more efficient activity [35,36].

## Discussion

For this *in vivo* study, we used the monoxenic mouse model in the same environmental conditions than for the *in vivo* transcriptional profiling performed previously with strain 630 [12]. This allows proceeding to some comparisons, which highlight the differences between the two strains regarding their adaptation to the host. As already observed with strain 630, genes of strain R20291 involved in metabolic functions, transport, stress response or coding for proteins with unknown functions, were the most regulated genes during the *in vivo* kinetic study.

The gut colonization by *C*. *difficile* is a prerequisite of the *C*. *difficile* infection process before toxin production [3]. We found that among genes encoding surface proteins in strain R20291, the adhesin CDR0802 was upregulated during the course of intestinal colonization (Table B in S1 File), which was not the case in strain 630 [12]. This difference may account for the better adherence of strain R20291 to the Caco-2 cells compared to strain 630 [37]. In contrast, the *slpA* gene encoding the S-layer proteins involved in in vitro adherence [4,38] was upregulated early in monoxenic mice for strain 630 [12] but not for strain R20291. These differences underline the relative importance of adhesins according to strains.

Our results showed that toxin genes were upregulated late, as already observed in strain 630 using the same animal model [12], but differ from those obtained in the pig ligated loop model where toxin production was induced early [39]. These differences are in agreement with the environmental conditions of the experiments. Indeed, in the pig ligated loop model the animals were fasted, which is consistent with the fact that toxin production is inversely correlated to nutrient availability. Importantly, we observed for the first time that the binary toxin genes are expressed during infection, supporting their role as potential virulence factors as already suggested [40].

In monoxenic mice, despite the imperfection of this model due to the absence of the competitive microbiota for the nutrient sources, the coordinate upregulation of genes involved in transport and metabolism of specific polysaccharides and amino acids may reflect the flexibility in nutrient acquisition by strain R20291. The upregulation of the PTS genes of cellobiose, a disaccharide produced by degradation of cellulose, and galactitol supports the notion that these sugars are present in the mouse digestive tract. Interestingly, several PTS-encoding genes were upregulated early or late during infection while others were downregulated such as ribose-specific PTS (CDR0303-0305) (Table D in S1 File). This may be explained either by the absence of these sugars in the gut content or by carbon sources being preferred over these substrates in strain R20291. Furthermore, we found that genes associated with the pentose phosphate pathway leading to the formation of glucolysis intermediates were induced in strain R20291 but not in strain 630. Also, whereas ethanolamine can be used as carbon and/or nitrogen sources in the gastro-intestinal lifestyle of strain 630 [12], in strain R20291, the *eut* operon (CDR1828-1846) involved in ethanolamine degradation was not regulated overtime indicating that ethanolamine is probably not used as a nutrient source by this strain. In addition, unlike strain 630, the *nagAB* genes (CDR0866-0867) coding for enzymes responsible for degradation of N-acetylglucosamine present in gastrointestinal mucins were upregulated at 14h in strain R20291. Thus the ability to use these different polysaccharides seems to indicate the capacity of the 027 strain to adapt its metabolism to the intestinal environment providing more advantages for colonization and multiplication compared to other bacteria. This is consistent with the in vitro competition assays in fecal bioreactors and in vivo studies in humanized microbiota mice, which recently demonstrated that PCR-ribotype 027 strains could outcompete strains from other ribotypes [41]. Thus these metabolic features associated with virulence factors and antibiotic resistance may participate in the epidemicity and the so-called hypervirulence of *C*. *difficile* 027 strains.

*C*. *difficile* can use amino acids as an energy source, through Stickland reactions [25]. Stickland reactions consist of the coupled fermentation of two amino acids in which one acts as an electron donor (leucine, isoleucine and alanine) while another acts as an electron acceptor (proline and glycine). In contrast to what was observed with strain 630, for which the leucine-proline appeared to be the Stickland pair preferentially used in vivo by this strain to generate ATP [12], no clear trends have evidenced suggesting that strain R20291 is able to use any pair in an equivalent way (Table E in S1 File).

**Fig 4:**
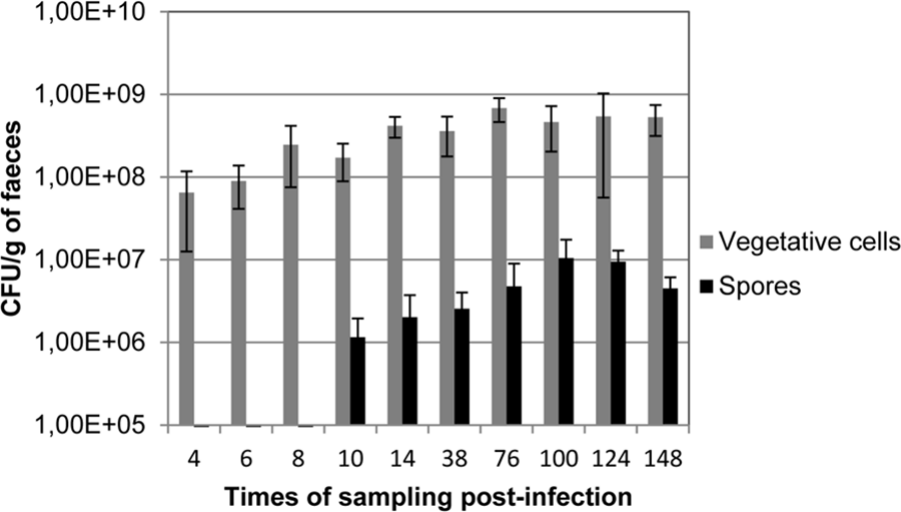
Kinetics of sporulation rate in *C*. *difficile* associated mice. Mice were orally challenged with 1x10^8^ CFUs of vegetative cells. Vegetative cells were enumerated on BHI agar plates and spores after a heat shock treatment on BHI containing 0.1% of taurocholate sodium salt.

Spores are the main forms of the contamination and dissemination of the *C*. *difficile* infection. Our transcriptional analysis clearly showed that spore production occurred in vivo in our monoxenic mouse model. Despite the lack of sporulation synchronization impeding data interpretation, our results suggest that part of vegetative cells set up in the mouse caeca are engaged rapidly in the sporulation process, as already observed for strain 630. For strain R20291 in this animal model, the sporulation process took place as early as 6h post-challenge. However, the sporulation rate (i.e. the ratio between spores and vegetative cells) measured for strain 630 was higher than for strain R20291: 50% versus 1% respectively at 38h post-infection (Fig 4). This sporulation rate is clearly in favor of vegetative cells for strain R20291, and this could contribute to the hypervirulence of the 027 strains [12].

Expression of sporulation encoding genes were also reported in the pig ligated loop, which replicates the more complex environment that *C*. *difficile* encounters during natural infection in humans [39]. This suggests that signals triggering sporulation are not exclusively associated with the microbiota activity or the lack of nutrient. The different sporulation rates between the R20291 and 630 strains observed in the same gut environment may reflect the different capacity of strains to sense the complex in vivo signals that promote *C*. *difficile* sporulation. Of note, the App transporter (CDR2558-2562) that has been shown to inhibit sporulation in *C*. *difficile* by facilitating the uptake of peptides and thus the availability to nutrients [42] was earlier and much more upregulated during infection in strain R20291 than in strain 630. This may account for the quite low sporulation rate observed in vivo in strain R20291.

Finally, several genes encoding proteins with unknown functions were differentially regulated in strain R20291 during the course of infection. Most of them are common with those already described in strain 630 [12] but interestingly their regulation are either similar or opposite (Table A in S1 File). For instance, CDR1478 (CD1581) encoding CdeM protein recently found to be associated to the exosporium layer in strain 630 [43] was highly upregulated late in R20291 and 630 strains. Similar upregulation in both strains were also observed for CDR0197 (CD0196), CDR0926 (CD1067) and CDR1962 (CD2055) genes, while some genes were more upregulated in strain R20291 such as CDR1511 (CD1613) and CDR3482 (CD3620). In addition, CDR0706-0709 and CDR2235 were only upregulated in strain R20291. Thus strain R20291 genes specifically upregulated during the infection process could participate in the hypervirulence of this 027 epidemic strain.

## Supporting Information

S1 File Tables A, B, C, D and E. (DOCX)
