# Supplementary material for: Deciphering Adaptation Strategies of the Epidemic Clostridium difficile 027 Strain during Infection through In Vivo Transcriptional Analysis
Source: PLoS One. 2016 Jun 28;11(6):e0158204. doi: 10.1371/journal.pone.0158204 (PMC4924792; doi:10.1371/journal.pone.0158204)
Supplement: S1 File — (DOCX) [file pone.0158204.s001.docx]

**Supporting information**

**S1 File: Tables A, B, C, D and E**

**Table A:** List of *C. difficile* R20291 genes differentially expressed (p<0.05) in *C. difficile* mono-associated mice as determined by microarrays analysis.

Genes up-regulated by a fold change of 1.5 or more are in red and those down-regulated by a fold change of 1.5 or more are in green

| **Gene ID CDR20291_** | **Gene Name** | **Description** | **Fold change compared to 8h** | | | | |
| --- | --- | --- | --- | --- | --- | --- | --- |
|  |  |  | **4H** | **6H** | **8H** | **14H** | **38H** |
| CDR0011 | *fusA1* | Elongation factor G (EF-G) | **0.15** | 1.00 | 1.00 | **0.44** | **0.19** |
| CDR0048 |  | Putative tRNA/rRNA methyltransferase. TrmH family. group3 | **2.62** | **1.96** | 1.00 | 1.00 | 1.00 |
| CDR0050 | *sigH* | RNA polymerase factor sigma-70 | **0.51** | 1.00 | 1.00 | 1.00 | **0.50** |
| CDR0053 | *secE* | Preprotein translocase SecE subunit | 1.00 | 1.00 | 1.00 | 1.00 | **1.95** |
| CDR0054 | *nusG* | transcription antitermination protein | 1.00 | 1.00 | 1.00 | 1.00 | **2.11** |
| CDR0055 | *rplK* | 50S ribosomal protein L11 | 1.00 | 1.00 | 1.00 | 1.00 | **2.19** |
| CDR0056 | *rplA* | 50S ribosomal protein L1 | 1.00 | 1.00 | 1.00 | 1.00 | **2.27** |
| CDR0057 | *rplJ* | 50S ribosomal protein L10 | 1.00 | 1.00 | 1.00 | 1.00 | **1.99** |
| CDR0058 | *rplL* | 50S ribosomal protein L7/L12 | 1.00 | 1.00 | 1.00 | 1.00 | **2.40** |
| CDR0059 |  | Putative NADP-dependent deshydrogenase | 1.00 | 1.00 | 1.00 | 1.00 | **0.52** |
| CDR0063 | *rpsG* | 30S ribosomal protein S7 | **2.06** | 1.00 | 1.00 | 1.00 | **2.02** |
| CDR0064 | *fusA* | Elongation factor G (EF-G) | 1.00 | 1.00 | 1.00 | 1.00 | **1.99** |
| CDR0066 | *rpsJ* | 30S ribosomal protein S10 | 1.00 | 1.00 | 1.00 | 1.00 | **2.18** |
| CDR0067 | *rplC* | 50S ribosomal protein L3 | **2.25** | 1.00 | 1.00 | 1.00 | **2.23** |
| CDR0068 | *rplD* | 50S ribosomal protein L4 | 1.00 | 1.00 | 1.00 | 1.00 | **2.04** |
| CDR0070 | *rplB* | 50S ribosomal protein L2 | 1.00 | 1.00 | 1.00 | 1.00 | **2.07** |
| CDR0071 | *rpsS* | 30S ribosomal protein S19 | **2.31** | 1.00 | 1.00 | 1.00 | **2.11** |
| CDR0072 | *rplV* | 50S ribosomal protein L22 | 1.00 | 1.00 | 1.00 | 1.00 | **2.26** |
| CDR0073 | *rpsC* | 30S ribosomal protein S3 | 1.00 | 1.00 | 1.00 | 1.00 | **2.10** |
| CDR0074 | *rplP* | 50S ribosomal protein L16 | **2.10** | 1.00 | 1.00 | 1.00 | **2.47** |
| CDR0075 | *rpmC* | 50S ribosomal protein L29 | **2.41** | 1.00 | 1.00 | 1.00 | **2.22** |
| CDR0076 | *rpsQ* | 30S ribosomal protein S17 | 1.00 | 1.00 | 1.00 | 1.00 | **2.31** |
| CDR0077 | *rplN* | 50S ribosomal protein L14 | **2.00** | 1.00 | 1.00 | 1.00 | **2.11** |
| CDR0078 | *rplX* | 50S ribosomal protein L24 | 1.00 | 1.00 | 1.00 | 1.00 | **2.52** |
| CDR0079 | *rplE* | 50S ribosomal protein L5 | 1.00 | 1.00 | 1.00 | 1.00 | **2.10** |
| CDR0080 | *rpsZ* | 30S ribosomal protein S14 type Z | 1.00 | 1.00 | 1.00 | 1.00 | **1.90** |
| CDR0082 | *rplF* | 50S ribosomal protein L6 | 1.00 | 1.00 | 1.00 | 1.00 | **2.08** |
| CDR0083 | *rplR* | 50S ribosomal protein L18 | 1.00 | 1.00 | 1.00 | 1.00 | **2.12** |
| CDR0085 | *rpmD* | 50S ribosomal protein L30 | 1.00 | 1.00 | 1.00 | 1.00 | **2.34** |
| CDR0087 | *prlA* | Preprotein translocase SecY subunit | **2.71** | 1.00 | 1.00 | 1.00 | **1.95** |
| CDR0088 | *adk* | Adenylate kinase | 1.00 | 1.00 | 1.00 | 1.00 | **1.93** |
| CDR0089 | *map1* | Methionine aminopeptidase Map1 (MAP) (Peptidase M) | 1.00 | 1.00 | 1.00 | 1.00 | **1.83** |
| CDR0090 |  | Ribosomal protein L14E/L6E/L27E-like | 1.00 | 1.00 | 1.00 | 1.00 | **2.19** |
| CDR0092 | *rpmJ* | 50S ribosomal protein L36 | 1.00 | 1.00 | 1.00 | 1.00 | **1.91** |
| CDR0103 | *rpsI* | 30S ribosomal protein S9 | **2.08** | 1.00 | 1.00 | 1.00 | **1.99** |
| CDR0104 | *cwlD* | Germination-specific N-acetylmuramoyl-L-alanine amidase. Autolysin | **0.19** | **0.16** | 1.00 | **0.35** | **0.16** |
| CDR0111 | *ptb* | phosphate butyryltransferase | 1.00 | 1.00 | 1.00 | 1.00 | **0.46** |
| CDR0112 | *buk* | Butyrate kinase (BK) (Branched-chain carboxylic acid kinase) | 1.00 | 1.00 | 1.00 | 1.00 | **0.46** |
| CDR0113 |  | Conserved hypothetical protein | 1.00 | 1.00 | 1.00 | 1.00 | **0.39** |
| CDR0114 |  | Putative 4Fe-4S ferredoxin. iron-sulfur binding domain protein. delta subunit | 1.00 | 1.00 | 1.00 | 1.00 | **0.38** |
| CDR0115 |  | Putative ferredoxin/flavodoxin oxidoreductase. alpha subunit | 1.00 | 1.00 | 1.00 | 1.00 | **0.36** |
| CDR0116 |  | Putative ferredoxin/flavodoxin oxidoreductase. beta subunit | 1.00 | 1.00 | 1.00 | 1.00 | **0.38** |
| CDR0117 |  | Putative ferredoxin/flavodoxin oxidoreductase. gamma subunit | **0.25** | 1.00 | 1.00 | 1.00 | **0.40** |
| CDR0118 | *glmM* | Phosphoglucosamine mutase | 1.00 | 1.00 | 1.00 | 1.00 | **2.40** |
| CDR0119 | *glmS* | Glucosamine--fructose-6-phosphate aminotransferase [isomerizing] | 1.00 | 1.00 | 1.00 | 1.00 | **2.33** |
| CDR0123 | *spoIID* | Stage II sporulation protein D | **0.42** | **0.24** | 1.00 | 1.00 | **0.45** |
| CDR0124 | *spoIIQ* | Stage II sporulation protein Q | **0.09** | **0.07** | 1.00 | **0.26** | **0.13** |
| CDR0125 | *spoIIID* | Stage III sporulation protein D | **0.11** | **0.12** | 1.00 | **0.32** | **0.15** |
| CDR0128 |  | Conserved hypothetical protein. DUF1256 family | **0.13** | **0.13** | 1.00 | **0.22** | **0.18** |
| CDR0130 |  | Putative membrane protein | **0.10** | **0.10** | 1.00 | 1.00 | **0.22** |
| CDR0135 |  | PTS system. lactose/cellobiose-family IIB component | **3.77** | 1.00 | 1.00 | 1.00 | **1.83** |
| CDR0138 |  | N(4)-(beta-N-acetylglucosaminyl)-L-asparaginase | **5.02** | 1.00 | 1.00 | 1.00 | **1.90** |
| CDR0146 |  | Putative transporter | 1.00 | 1.00 | 1.00 | **0.50** | 1.00 |
| CDR0163 |  | Conserved hypothetical protein | **0.05** | 1.00 | 1.00 | 1.00 | **0.49** |
| CDR0172 |  | Conserved hypothetical protein | **0.48** | 1.00 | 1.00 | 1.00 | **0.54** |
| CDR0180 | *gluD* | NAD-specific glutamate dehydrogenase | 1.00 | 1.00 | 1.00 | 1.00 | **0.39** |
| CDR0197 |  | Fragment of conserved hypothetical protein. DUF111 family (part 2) | 1.00 | 1.00 | 1.00 | **6.53** | **3.48** |
| CDR0199 |  | Putative membrane-associated nucleotidase | 1.00 | **2.19** | 1.00 | 1.00 | 1.00 |
| CDR0215 | *purE* | Phosphoribosylaminoimidazole carboxylase catalytic subunit | 1.00 | 1.00 | 1.00 | 1.00 | **2.65** |
| CDR0216 | *purC* | Phosphoribosylaminoimidazole-succinocarboxamide synthase | 1.00 | 1.00 | 1.00 | 1.00 | **2.61** |
| CDR0217 | *purF* | Amidophosphoribosyltransferase | 1.00 | 1.00 | 1.00 | 1.00 | **3.88** |
| CDR0218 | *purG* | Phosphoribosylformylglycinamidine cyclo-ligase | 1.00 | 1.00 | 1.00 | 1.00 | **3.49** |
| CDR0219 | *purN* | Phosphoribosylglycinamide formyltransferase | 1.00 | 1.00 | 1.00 | 1.00 | **4.26** |
| CDR0220 | *purH* | Bifunctional purine biosynthesis protein purH [Includes: Phosphoribosylaminoimidazolecarboxamide formyltransferase IMP cyclohydrolase] | 1.00 | 1.00 | 1.00 | 1.00 | **3.92** |
| CDR0221 | *purD* | Phosphoribosylamine--glycine ligase | 1.00 | 1.00 | 1.00 | 1.00 | **2.84** |
| CDR0222 | *purL* | Phosphoribosylformylglycinamidine synthase | 1.00 | 1.00 | 1.00 | 1.00 | **3.41** |
| CDR0259 | *FlbD* | Flagellar protein FlbD | 1.00 | 1.00 | 1.00 | **2.01** | **1.79** |
| CDR0261 | *motB* | Flagellar motor rotation protein MotB (Chemotaxis protein MotB) | 1.00 | 1.00 | 1.00 | **2.08** | **1.94** |
| CDR0264 | *fliP* | Flagellar biosynthesis protein FliP | 1.00 | 1.00 | 1.00 | 1.00 | **2.03** |
| CDR0271 |  | Putative flagellar protein | **2.69** | 1.00 | 1.00 | 1.00 | **1.91** |
| CDR0293 |  | Conserved hypothetical protein | **2.43** | **2.58** | 1.00 | 1.00 | 1.00 |
| CDR0303 | *rbsB* | ABC-type transport system. ribose-specific extracellular solute-binding protein | **0.24** | **0.35** | 1.00 | **0.36** | **0.29** |
| CDR0304 | *rbsA* | ABC-type transport system. ribose-specific ATP-binding protein | 1.00 | **0.30** | 1.00 | **0.36** | **0.27** |
| CDR0305 | *rbsC* | ABC-type transport system. ribose-specific permease | **0.25** | **0.28** | 1.00 | **0.46** | **0.36** |
| CDR0306 | *argE* | Acetylornithine deacetylase ArgE | **0.28** | **0.36** | 1.00 | **0.32** | **0.28** |
| CDR0307 |  | Conserved hypothetical protein. DUF1355 family | 1.00 | 1.00 | 1.00 | **0.42** | **0.33** |
| CDR0314 |  | Putative hydrolase. HAD superfamily. subfamily IB | 1.00 | 1.00 | 1.00 | **2.37** | 1.00 |
| CDR0316 |  | Conserved hypothetical protein | **0.04** | **0.03** | 1.00 | 1.00 | **0.13** |
| CDR0319 |  | Putative membrane protein | **0.42** | **0.39** | 1.00 | **0.37** | **0.39** |
| CDR0329 | *cbiM* | Cobalamin biosynthesis protein | **0.33** | **0.35** | 1.00 | 1.00 | **0.32** |
| CDR0330 | *cbiN* | ABC-type transport system. cobalt-specific extracellular solute-binding protein | **0.35** | **0.37** | 1.00 | 1.00 | **0.31** |
| CDR0332 | *cbiO* | ABC-type transport system. cobalt-specific ATP-binding protein | 1.00 | 1.00 | 1.00 | 1.00 | **0.48** |
| CDR0337 |  |  | 1.00 | 1.00 | 1.00 | **2.46** | 1.00 |
| CDR0353 |  | hypothetical protein | 1.00 | **0.30** | 1.00 | **0.44** | **0.50** |
| CDR0386 | *ortA* | 2-amino-4-ketopentanoate thiolase alpha subunit | **0.38** | **0.42** | 1.00 | **0.31** | 1.00 |
| CDR0387 | *ortB* | 2-amino-4-ketopentanoate thiolase beta subunit | **0.37** | 1.00 | 1.00 | **0.29** | 1.00 |
| CDR0388 | *oraS* | D-ornithine aminomutase S component | **0.28** | **0.30** | 1.00 | **0.22** | **0.26** |
| CDR0389 | *oraE* | D-ornithine aminomutase E component | **0.17** | **0.18** | 1.00 | **0.22** | **0.27** |
| CDR0390 |  | Putative reactivating factor for adenosylcobalamine-dependent D-ornithine aminomutase | **0.26** | **0.32** | 1.00 | **0.21** | **0.24** |
| CDR0392 |  | Na+/H+ antiporter. NhaA family | 1.00 | 1.00 | 1.00 | **0.29** | **0.29** |
| CDR0432 |  | Putative phosphoribosylaminoimidazole-succinocarboxamide synthetase | 1.00 | 1.00 | 1.00 | 1.00 | **3.18** |
| CDR0445 |  | Conserved hypothetical protein | 1.00 | **2.03** | 1.00 | 1.00 | **0.46** |
| CDR0451 |  | Fragment of aminobenzoyl-glutamate transporter (Part 1) | **2.50** | **2.13** | 1.00 | 1.00 | 1.00 |
| CDR0474 |  | Conserved hypothetical protein | 1.00 | 1.00 | 1.00 | **2.39** | 1.00 |
| CDR0476 | *sleC* | Spore exo-acting lytic transglycosylase | **0.14** | **0.15** | 1.00 | 1.00 | 1.00 |
| CDR0479 | *sip2* | Signal peptidase type I | **0.37** | 1.00 | 1.00 | 1.00 | **0.46** |
| CDR0480 | *sip2* | Signal peptidase type I | **0.39** | 1.00 | 1.00 | 1.00 | **0.44** |
| CDR0482 |  | Putative uridine kinase | **0.21** | **0.33** | 1.00 | 1.00 | **0.29** |
| CDR0489 |  | putative ATP-dependent protease | 1.00 | **0.42** | 1.00 | 1.00 | 1.00 |
| CDR0495 |  | Conserved hypothetical protein | **0.20** | **0.35** | 1.00 | 1.00 | **0.27** |
| CDR0496 |  | Putative sporulation protein | **0.27** | **0.37** | 1.00 | 1.00 | **0.28** |
| CDR0497 |  | Putative membrane protein | **0.20** | **0.35** | 1.00 | 1.00 | **0.26** |
| CDR0507 | *gapN* | Glyceraldehyde-3-phosphate dehydrogenase (NADP(+)) (GADPH) | 1.00 | 1.00 | 1.00 | **0.48** | 1.00 |
| CDR0510 |  | signaling protein | 1.00 | 1.00 | 1.00 | **2.64** | 1.00 |
| CDR0511 |  | Conserved hypothetical protein | **0.25** | 1.00 | 1.00 | **0.19** | **0.16** |
| CDR0512 |  | Conserved hypothetical protein | 1.00 | 1.00 | 1.00 | **0.20** | **0.18** |
| CDR0521 |  | Conserved hypothetical protein | 1.00 | 1.00 | 1.00 | **72.50** | **54.30** |
| CDR0522 | *cotF* | Spore coat protein | 1.00 | 1.00 | 1.00 | **74.91** | **66.46** |
| CDR0523 | *cotCB* | Spore-coat protein CotCB manganese catalase | 1.00 | 1.00 | 1.00 | **66.46** | **59.32** |
| CDR0552 |  | Transcriptional regulator. Crp family | **0.31** | **0.32** | 1.00 | **0.42** | **0.27** |
| CDR0582 | *tcdB* | Toxin B | 1.00 | 1.00 | 1.00 | 1.00 | **4.53** |
| CDR0583 | *tcdE* | Holin-like pore-forming protein | 1.00 | 1.00 | 1.00 | 1.00 | **1.96** |
| CDR0584 | *tcdA* | Toxin A | **0.47** | 1.00 | 1.00 | 1.00 | **2.87** |
| CDR0587 |  | Conserved hypothetical protein | 1.00 | 1.00 | 1.00 | 1.00 | **2.28** |
| CDR0588 | *cdd2* | ABC-type transport system. lantibiotic/multidrug-family permease | 1.00 | 1.00 | 1.00 | **2.49** | **2.20** |
| CDR0589 | *cdd3* | ABC-type transport system. lantibiotic/multidrug-family permease | 1.00 | 1.00 | 1.00 | 1.00 | **2.12** |
| CDR0590 | *cdd4* | ABC-type transport system. lantibiotic/multidrug-family ATP-binding protein | 1.00 | 1.00 | 1.00 | **2.58** | **2.82** |
| CDR0595 |  |  | 1.00 | **2.60** | 1.00 | 1.00 | 1.00 |
| CDR0598 |  | Putative acetyltransferase | 1.00 | 1.00 | 1.00 | **4.34** | **3.17** |
| CDR0610 |  | Putative ATP-dependent peptidase. M41 family | **0.19** | **0.21** | 1.00 | 1.00 | **0.24** |
| CDR0611 | *infC* | Translation initiation factor IF-3 | **2.01** | 1.00 | 1.00 | 1.00 | **2.12** |
| CDR0613 | *rplT* | 50S ribosomal protein L20 | 1.00 | 1.00 | 1.00 | 1.00 | **1.79** |
| CDR0616 |  | Putative nucleotide phosphodiesterase | **0.40** | **0.44** | 1.00 | 1.00 | **0.44** |
| CDR0617 |  | Putative short chain dehydrogenase/reductase. SDR family | **0.30** | **0.25** | 1.00 | **0.29** | 1.00 |
| CDR0621 |  | Putative exported metalloendopeptidase | **0.49** | 1.00 | 1.00 | **0.48** | 1.00 |
| CDR0642 | *ptb* | Phosphate butyryltransferase (Phosphotransbutyrylase) | 1.00 | 1.00 | 1.00 | 1.00 | **0.47** |
| CDR0643 | *cooS* | Bifunctional carbon monoxide dehydrogenase/acetyl-CoA synthase (CODH) | **0.17** | 1.00 | 1.00 | 1.00 | **0.44** |
| CDR0645 | *fhs* | Formate--tetrahydrofolate ligase (Formyltetrahydrofolate synthetase) (FHS) (FTHFS) | **0.27** | 1.00 | 1.00 | 1.00 | **0.46** |
| CDR0661 |  | Conserved hypothetical protein | 1.00 | 1.00 | 1.00 | 1.00 | **0.50** |
| CDR0663 |  | Conserved hypothetical protein | 1.00 | 1.00 | 1.00 | 1.00 | **0.38** |
| CDR0673 |  | Putative OmpA/MotB proton channel | **2.35** | 1.00 | 1.00 | **2.43** | **2.17** |
| CDR0677 |  | Putative DNA helicase. UvrD/REP type | 1.00 | 1.00 | 1.00 | **5.93** | **4.12** |
| CDR0687 | *plfB* | Formate acetyltransferase (Pyruvate formate-lyase) | **5.56** | 1.00 | 1.00 | 1.00 | **2.25** |
| CDR0688 |  | Putative Ca2+/Na+ antiporter | **0.19** | **0.17** | 1.00 | **0.31** | **0.10** |
| CDR0699 | *spoIIAA* | Anti-sigma F factor antagonist | **0.08** | 1.00 | 1.00 | 1.00 | **0.26** |
| CDR0700 | *spoIIAB* | Anti-sigma F factor (Stage II sporulation protein AB) | **0.10** | 1.00 | 1.00 | 1.00 | **0.28** |
| CDR0701 | *sigF* | RNA polymerase sigma-F factor | **0.20** | 1.00 | 1.00 | 1.00 | **0.31** |
| CDR0702 | *spoVAC* | Stage V sporulation protein AC | **0.24** | **0.26** | 1.00 | 1.00 | 1.00 |
| CDR0703 | *spoVAD* | Stage V sporulation protein AD | **0.31** | **0.29** | 1.00 | 1.00 | **0.33** |
| CDR0704 | *spoVAE* | Stage V sporulation protein AE | **0.39** | **0.43** | 1.00 | 1.00 | 1.00 |
| CDR0706 |  | Putative membrane protein | **3.43** | 1.00 | 1.00 | **3.33** | **2.58** |
| CDR0707 |  | Conserved hypothetical protein | **3.95** | 1.00 | 1.00 | **4.10** | **3.18** |
| CDR0708 |  | Putative amidohydrolase. M20D peptidase family | **4.91** | 1.00 | 1.00 | **3.64** | **2.73** |
| CDR0709 |  | Conserved hypothetical protein. DUF1177 family | **3.12** | 1.00 | 1.00 | **2.84** | **2.16** |
| CDR0714 | *spoIVB* | Stage IV sporulation protein SpoIVB. S55 peptidase family | 1.00 | 1.00 | 1.00 | 1.00 | **3.05** |
| CDR0715 |  | Putative N-acetylmuramoyl-L-alanine amidase | **0.20** | **0.19** | 1.00 | **0.40** | **0.26** |
| CDR0719 |  | Putative GTP-binding protein. HflX type | **0.29** | 1.00 | 1.00 | 1.00 | **0.41** |
| CDR0720 |  | Conserved hypothetical protein | **0.27** | 1.00 | 1.00 | 1.00 | **0.35** |
| CDR0721 |  | Putative hydrolase. NUDIX family | **0.36** | 1.00 | 1.00 | 1.00 | **0.36** |
| CDR0723 |  | Putative membrane protein. DUF81 family | **0.30** | **0.34** | 1.00 | 1.00 | **0.34** |
| CDR0724 |  | Putative membrane protein. DUF81 family | **0.30** | **0.32** | 1.00 | 1.00 | **0.30** |
| CDR0734 |  | Putative acyl-CoA dehydrogenase | 1.00 | 1.00 | 1.00 | **0.49** | **0.41** |
| CDR0735 | *etfB* | Electron transfer flavoprotein beta-subunit. core | **0.48** | 1.00 | 1.00 | **0.50** | **0.47** |
| CDR0736 | *etfA* | Electron transfer flavoprotein alpha-subunit | **0.43** | 1.00 | 1.00 | **0.42** | **0.39** |
| CDR0738 |  | Conserved hypothetical protein | 1.00 | 1.00 | 1.00 | **0.49** | 1.00 |
| CDR0743 |  | Putative universal stress protein A (UspA) | 1.00 | 1.00 | 1.00 | 1.00 | **0.52** |
| CDR0755 | *rbr* | Rubrerythrin | 1.00 | 1.00 | 1.00 | 1.00 | **0.48** |
| CDR0756 |  | Putative ferric-uptake regulator | 1.00 | 1.00 | 1.00 | **0.51** | **0.46** |
| CDR0757 | *rbo* | Rubredoxin oxidoreductase (desulfoferrodoxin) | **0.38** | 1.00 | 1.00 | 1.00 | **0.50** |
| CDR0768 |  | Putative DNA-binding protein | **0.38** | 1.00 | 1.00 | 1.00 | **0.47** |
| CDR0769 |  | Putative membrane protein | **0.29** | 1.00 | 1.00 | 1.00 | **0.43** |
| CDR0770 |  | Putative isomerase/hydrolase | **0.36** | 1.00 | 1.00 | 1.00 | **0.35** |
| CDR0794 |  | Putative ADP-ribose binding protein | 1.00 | 1.00 | 1.00 | 1.00 | **0.50** |
| CDR0812 | *glgC* | Glucose-1-phosphate adenylyltransferase | **0.24** | 1.00 | 1.00 | 1.00 | **0.34** |
| CDR0813 | *glgD* | Glycogen biosynthesis protein | **0.30** | 1.00 | 1.00 | 1.00 | **0.39** |
| CDR0814 | *glgA* | Glycogen synthase (Starch [bacterial glycogen] synthase) | **0.32** | 1.00 | 1.00 | 1.00 | **0.42** |
| CDR0815 | *glgP* | Glycogen phosphorylase | **0.27** | 1.00 | 1.00 | 1.00 | **0.38** |
| CDR0816 |  | Putative alpha-amylase | **0.28** | 1.00 | 1.00 | 1.00 | **0.38** |
| CDR0830 | *opuCA* | ABC-type transport system. glycine betaine/carnitine/choline ATP-binding protein | 1.00 | **2.14** | 1.00 | 1.00 | **0.43** |
| CDR0831 | *opuCC* | ABC-type transport system. glycine betaine/carnitine/choline permease | 1.00 | **2.38** | 1.00 | 1.00 | **0.46** |
| CDR0848 | *serA* | Putative D-3-phosphoglycerate dehydrogenase | 1.00 | **0.43** | 1.00 | **0.42** | **0.42** |
| CDR0849 |  | Conserved hypothetical protein | 1.00 | **0.30** | 1.00 | 1.00 | **0.40** |
| CDR0865 |  | Transcriptional regulator. GntR family | 1.00 | **2.51** | 1.00 | **3.09** | 1.00 |
| CDR0866 | *nagA* | N-acetylglucosamine-6-phosphate deacetylase (GlcNAc 6-P deacetylase) | 1.00 | 1.00 | 1.00 | **2.82** | 1.00 |
| CDR0867 | *nagB* | Glucosamine-6-phosphate deaminase | 1.00 | 1.00 | 1.00 | **2.68** | 1.00 |
| CDR0868 |  | Putative membrane protein | **0.38** | 1.00 | 1.00 | 1.00 | **0.48** |
| CDR0910 | *bcd2* | Butyryl-CoA dehydrogenase | 1.00 | 1.00 | 1.00 | 1.00 | **0.53** |
| CDR0912 | *etfA* | Electron transfer flavoprotein subunit alpha | 1.00 | 1.00 | 1.00 | 1.00 | **0.49** |
| CDR0913 | *crt2* | 3-hydroxybutyryl-CoA dehydratase (Crotonase) | 1.00 | 1.00 | 1.00 | 1.00 | **0.54** |
| CDR0914 | *hbd* | 3-hydroxybutyryl-CoA dehydrogenase | 1.00 | 1.00 | 1.00 | 1.00 | **0.46** |
| CDR0915 | *thlA1* | Acetoacetyl-CoA thiolase 1 | **0.16** | 1.00 | 1.00 | 1.00 | **0.48** |
| CDR0919 |  | Conserved hypothetical protein | **0.21** | **0.23** | 1.00 | **0.33** | **0.24** |
| CDR0920 |  | Conserved hypothetical protein | **0.19** | **0.08** | 1.00 | **3.38** | **3.78** |
| CDR0921 |  | Conserved hypothetical protein | **0.03** | **0.03** | 1.00 | 1.00 | 1.00 |
| CDR0922 |  | Conserved hypothetical protein | **0.02** | **0.01** | 1.00 | 1.00 | 1.00 |
| CDR0924 |  | Conserved hypothetical protein | **0.02** | **0.02** | 1.00 | 1.00 | 1.00 |
| CDR0925 |  | Conserved hypothetical protein | **0.22** | **0.36** | 1.00 | 1.00 | 1.00 |
| CDR0926 | *CdeC* | Clostridium difficile exosporium cycteine-rich protein | **0.10** | **0.08** | 1.00 | **10.15** | **7.42** |
| CDR0927 |  | Putative polysaccharide biosynthesis/sporulation protein | **0.23** | **0.20** | 1.00 | 1.00 | **0.48** |
| CDR0957 |  | Nitroreductase-family protein | 1.00 | 1.00 | 1.00 | **0.53** | **0.51** |
| CDR0964 | *polA* | DNA polymerase I (POLI) | **0.32** | **0.41** | 1.00 | **0.43** | **0.32** |
| CDR0965 | *coaE* | Dephospho-CoA kinase (Dephosphocoenzyme A kinase) | **0.48** | **0.40** | 1.00 | 1.00 | **0.46** |
| CDR0967 |  | Putative solute-binding lipoprotein | **0.26** | **0.25** | 1.00 | 1.00 | **0.34** |
| CDR0968 |  | Putative heavy-metal transport/detoxification protein | 1.00 | **0.42** | 1.00 | 1.00 | 1.00 |
| CDR0969 |  | Conserved hypothetical protein | **0.12** | **0.11** | 1.00 | **2.59** | 1.00 |
| CDR0972 |  | Conserved hypothetical protein | **0.23** | **0.19** | 1.00 | **0.49** | **0.16** |
| CDR0986 | *minC* | Cell division regulator (septum placement) | **0.33** | 1.00 | 1.00 | 1.00 | **0.49** |
| CDR0987 | *minD* | Septum site-determining protein MinD (Cell division inhibitor MinD) | **0.34** | 1.00 | 1.00 | 1.00 | **0.54** |
| CDR0988 | *minE* | Cell division topological specificity factor | **0.24** | 1.00 | 1.00 | 1.00 | **0.43** |
| CDR1004 | *recV* | Tyrosine DNA recombinase. XerC/XerD family | **0.46** | **0.43** | 1.00 | **0.45** | **0.45** |
| CDR1005 |  | Putative membrane protein | **0.28** | **0.25** | 1.00 | **0.40** | **0.33** |
| CDR1030 | *spoIIIAA* | Stage III sporulation protein AA | **0.14** | **0.26** | 1.00 | **0.34** | **0.15** |
| CDR1031 | *spoIIIAB* | Stage III sporulation protein AB | **0.22** | **0.20** | 1.00 | **0.30** | **0.28** |
| CDR1032 | *spoIIIAC* | Stage III sporulation protein AC | **0.20** | **0.20** | 1.00 | **0.26** | **0.22** |
| CDR1033 | *spoIIIAD* | Stage III sporulation protein AD | **0.18** | **0.18** | 1.00 | **0.24** | **0.17** |
| CDR1034 | *spoIIIAE* | Stage III sporulation protein AE | **0.23** | **0.24** | 1.00 | **0.27** | **0.22** |
| CDR1035 | *spoiIIIAF* | Stage III sporulation protein AF | **0.30** | **0.27** | 1.00 | **0.34** | **0.34** |
| CDR1036 | *spoIIIAG* | Stage III sporulation protein AG | **0.05** | **0.06** | 1.00 | **0.26** | **0.09** |
| CDR1037 | *spoIIIAH* | Stage III sporulation protein AH | **0.06** | **0.07** | 1.00 | **0.26** | **0.10** |
| CDR1052 | *spo0A* | Stage 0 sporulation protein A | **0.25** | 1.00 | 1.00 | 1.00 | **0.40** |
| CDR1063 | *pdp* | Pyrimidine-nucleoside phosphorylase | 1.00 | **0.47** | 1.00 | 1.00 | **0.51** |
| CDR1065 |  | Putative O-methyltransferase | **2.95** | **2.10** | 1.00 | 1.00 | 1.00 |
| CDR1067 |  | Putative peptidoglycan glycosyltransferase | **0.42** | **0.40** | 1.00 | **0.39** | **0.41** |
| CDR1073 |  | Putative phage protein | **0.31** | **0.36** | 1.00 | 1.00 | **0.40** |
| CDR1094 |  | Putative RNA-binding protein | 1.00 | 1.00 | 1.00 | 1.00 | **2.04** |
| CDR1097 | *rplS* | 50S ribosomal protein L19 | 1.00 | 1.00 | 1.00 | 1.00 | **2.44** |
| CDR1099 | *brnQ-1* | Branched chain amino acid transport system carrier protein | **0.10** | 1.00 | 1.00 | 1.00 | **0.21** |
| CDR1127 | *fur* | Transcriptional regulator. Fur family | 1.00 | 1.00 | 1.00 | 1.00 | **0.52** |
| CDR1128 |  | Putative membrane protein | 1.00 | 1.00 | 1.00 | **2.44** | 1.00 |
| CDR1131 | *dacF* | D-alanyl-D-alanine carboxypeptidase | **0.19** | **0.21** | 1.00 | 1.00 | 1.00 |
| CDR1137 |  | Conserved hypothetical protein | **0.36** | **0.33** | 1.00 | **0.41** | **0.30** |
| CDR1138 |  | Conserved hypothetical protein | **0.17** | **0.17** | 1.00 | **0.29** | **0.18** |
| CDR1141 |  | Putative membrane protein | **0.40** | **0.38** | 1.00 | 1.00 | **0.31** |
| CDR1142 |  | Conserved hypothetical protein | **0.34** | **0.35** | 1.00 | 1.00 | 1.00 |
| CDR1160 |  | Putative polysaccharide deacetylase | **0.26** | **0.33** | 1.00 | 1.00 | **0.25** |
| CDR1161 |  | Putative peptidase. M16 family | **0.37** | **0.41** | 1.00 | 1.00 | **0.44** |
| CDR1162 |  | Putative sporulation protein | **0.26** | **0.36** | 1.00 | 1.00 | **0.37** |
| CDR1163 | *dapG* | Aspartokinase 1 (Aspartokinase I) (1spartate kinase I) | **0.27** | **0.36** | 1.00 | 1.00 | **0.40** |
| CDR1164 | *tepA* | Protein export-enhancing factor | **0.28** | **0.32** | 1.00 | 1.00 | **0.42** |
| CDR1171 |  | Putative DNA helicase. UvrD/REP type | **0.43** | 1.00 | 1.00 | 1.00 | **0.45** |
| CDR1180 | *aspB* | Aspartate aminotransferase (AspAT) (Transaminase A) | 1.00 | 1.00 | 1.00 | 1.00 | **0.45** |
| CDR1196 |  | Conserved hypothetical protein | **0.41** | **0.36** | 1.00 | 1.00 | 1.00 |
| CDR1198 |  | Putative CBS domain-containing protein | **0.44** | **0.43** | 1.00 | 1.00 | **0.47** |
| CDR1199 |  | Putative peptidyl-prolyl isomerase | 1.00 | 1.00 | 1.00 | 1.00 | **1.90** |
| CDR1242 |  | Putative amino acid amidase | **0.51** | 1.00 | 1.00 | 1.00 | **0.47** |
| CDR1243 |  | Conserved hypothetical protein. UPF0066 family | 1.00 | 1.00 | 1.00 | 1.00 | **0.42** |
| CDR1244 |  | Putative peptidase. M20D family | **0.41** | **0.40** | 1.00 | **0.39** | **0.38** |
| CDR1247 |  | Putative isochorismatase | 1.00 | 1.00 | 1.00 | 1.00 | **0.43** |
| CDR1248 | *glpQ* | Glycerophosphoryl diester phosphodiesterase | **0.32** | **0.30** | 1.00 | 1.00 | 1.00 |
| CDR1249 |  | Putative synthetase | **0.26** | **0.33** | 1.00 | 1.00 | **0.27** |
| CDR1250 |  | Putative oligopeptide transporter | **0.41** | 1.00 | 1.00 | 1.00 | **0.46** |
| CDR1251 |  | Conserved hypothetical protein | 1.00 | 1.00 | 1.00 | 1.00 | **0.45** |
| CDR1263 |  | Putative membrane protein | **0.32** | **0.32** | 1.00 | **0.31** | **0.27** |
| CDR1264 |  | Putative ATP-binding protein | 1.00 | 1.00 | 1.00 | 1.00 | **0.43** |
| CDR1271 |  | Conserved hypothetical protein | **0.25** | 1.00 | 1.00 | 1.00 | **0.40** |
| CDR1279 | *pdaA* | Exported N-acetylmuramic acid deacetylase | **0.30** | **0.28** | 1.00 | 1.00 | 1.00 |
| CDR1282 | *cotE* | Spore coat protein CotE peroxiredoxin/chitinase | **0.07** | **0.07** | 1.00 | **3.76** | 1.00 |
| CDR1312 |  | Conserved hypothetical protein | **0.21** | **0.24** | 1.00 | 1.00 | 1.00 |
| CDR1320 |  | Putative membrane protein | 1.00 | **0.44** | 1.00 | 1.00 | 1.00 |
| CDR1326 | *feoA* | Ferrous iron transport protein | 1.00 | 1.00 | 1.00 | **0.46** | **0.23** |
| CDR1327 | *feoA* | Ferrous iron transport protein | 1.00 | 1.00 | 1.00 | 1.00 | **0.43** |
| CDR1328 | *feoB1* | Ferrous iron transport protein B | **2.21** | 1.00 | 1.00 | 1.00 | **0.38** |
| CDR1329 |  | Conserved hypothetical protein | 1.00 | 1.00 | 1.00 | **0.45** | **0.35** |
| CDR1334 |  | Conserved hypothetical protein | 1.00 | 1.00 | 1.00 | **0.29** | **0.19** |
| CDR1335 |  | Putative ribosome recycling factor | **0.18** | **0.22** | 1.00 | 1.00 | 1.00 |
| CDR1354 |  | Putative drug/sodium antiporter. MATE family | 1.00 | 1.00 | 1.00 | 1.00 | **2.21** |
| CDR1359 |  | Conserved hypothetical protein. DUF1121 family | **0.47** | 1.00 | 1.00 | 1.00 | **0.42** |
| CDR1360 | *cotB* | Spore outer coat layer protein CotB | **0.06** | **0.08** | 1.00 | **0.40** | **0.14** |
| CDR1361 | *panC* | Pantothenate synthetase | 1.00 | 1.00 | 1.00 | **3.73** | 1.00 |
| CDR1362 | *panB* | Ketopantoate hydroxymethyltransferase | 1.00 | 1.00 | 1.00 | **3.75** | 1.00 |
| CDR1363 | *panG* | Putative ketopantoate reductase | 1.00 | 1.00 | 1.00 | **3.68** | 1.00 |
| CDR1366 | *feoB* | Ferrous iron transport protein B | **0.37** | 1.00 | 1.00 | **3.32** | 1.00 |
| CDR1367 | *feoA* | Ferrous iron transport protein | **0.40** | 1.00 | 1.00 | **2.96** | 1.00 |
| CDR1372 |  | Putative transporter | 1.00 | 1.00 | 1.00 | 1.00 | **2.08** |
| CDR1385 |  | Ferredoxin--NADP(+) reductase subunit alpha | 1.00 | 1.00 | 1.00 | 1.00 | **0.38** |
| CDR1386 | *aspB* | Putative glutamate synthase | 1.00 | 1.00 | 1.00 | 1.00 | **0.46** |
| CDR1393 |  | Conserved hypothetical protein | **0.25** | 1.00 | 1.00 | 1.00 | **0.24** |
| CDR1404 |  | Putative amino acid permease | 1.00 | **0.35** | 1.00 | 1.00 | 1.00 |
| CDR1465 | *cotG* | Putative spore-associated manganese catalase | **0.26** | **0.33** | 1.00 | 1.00 | **0.31** |
| CDR1466 |  | Conserved hypothetical protein | 1.00 | 1.00 | 1.00 | 1.00 | **0.54** |
| CDR1472 |  | Conserved hypothetical protein | **0.19** | **0.21** | 1.00 | **0.35** | **0.26** |
| CDR1478 |  | Conserved hypothetical protein | 1.00 | 1.00 | 1.00 | **110.47** | **156.50** |
| CDR1488 |  | Putative membrane protein | **0.31** | 1.00 | 1.00 | 1.00 | **0.44** |
| CDR1501 |  | ABC-type transport system. multidrug-family permease | 1.00 | 1.00 | 1.00 | **2.93** | **2.73** |
| CDR1502 |  | ABC-type transport system. multidrug-family ATP-binding protein | 1.00 | 1.00 | 1.00 | **2.67** | **2.59** |
| CDR1503 |  | Putative transcriptional regulator | 1.00 | 1.00 | 1.00 | **2.94** | **2.38** |
| CDR1511 | *cotA* | Spore outer coat layer protein CotA | 1.00 | 1.00 | 1.00 | **19.35** | **15.89** |
| CDR1520 |  | Putative lipoprotein | **0.30** | 1.00 | 1.00 | 1.00 | **0.48** |
| CDR1521 |  | Putative oxidoreductase | **0.23** | **0.34** | 1.00 | 1.00 | **0.48** |
| CDR1529 | *sodA* | Spore coat protein. superoxide dismutase | **0.26** | **0.27** | 1.00 | 1.00 | 1.00 |
| CDR1592 |  | Conserved hypothetical protein | **0.32** | 1.00 | 1.00 | 1.00 | **0.37** |
| CDR1606 |  | Putative C4-dicarboxylate anaerobic carrier. DcuC family | **0.27** | **0.27** | 1.00 | 1.00 | 1.00 |
| CDR1610 |  | Conserved hypothetical protein. UPF0145 family | **0.37** | 1.00 | 1.00 | 1.00 | **0.44** |
| CDR1623 |  | Conserved hypothetical protein | **0.09** | **0.09** | 1.00 | **0.18** | **0.10** |
| CDR1637 |  | Glycine/sarcosine/betaine reductase complex. protein B. alpha and beta subunits | **0.39** | 1.00 | 1.00 | **0.39** | **0.32** |
| CDR1639 | *feoA* | Ferrous iron transport protein | 1.00 | 1.00 | 1.00 | 1.00 | **0.34** |
| CDR1643 |  | Putative 2-hydroxyacyl-CoA dehydratase | 1.00 | 1.00 | 1.00 | **2.07** | **2.42** |
| CDR1644 |  | Putative CoA enzyme activase | 1.00 | 1.00 | 1.00 | **2.14** | **2.32** |
| CDR1662 | *gapB* | Glyceraldehyde-3-phosphate dehydrogenase (GAPDH) | 1.00 | 1.00 | 1.00 | **0.30** | **0.16** |
| CDR1664 |  | Putative CoA enzyme activase | 1.00 | 1.00 | 1.00 | 1.00 | **0.46** |
| CDR1683 |  | Conserved hypothetical protein | **0.08** | **0.10** | 1.00 | 1.00 | **0.15** |
| CDR1688 |  | Conserved hypothetical protein | 1.00 | 1.00 | 1.00 | **0.35** | **0.27** |
| CDR1718 |  | P-type calcium transport ATPase | **0.39** | **0.40** | 1.00 | 1.00 | **0.58** |
| CDR1739 |  |  | 1.00 | **0.44** | 1.00 | 1.00 | 1.00 |
| CDR1740 |  | Fragment of putative methyltransferase (N-terminal region) | 1.00 | 1.00 | 1.00 | **16.12** | **9.86** |
| CDR1741 |  | Putative membrane protein Tn1549-like. CTn5-Orf1 | 1.00 | 1.00 | 1.00 | **5.55** | **3.73** |
| CDR1781 |  | hypothetical protein | 1.00 | 1.00 | 1.00 | 1.00 | **2.38** |
| CDR1784 |  | hypothetical protein | 1.00 | 1.00 | 1.00 | 1.00 | **3.26** |
| CDR1786 |  | hypothetical protein | 1.00 | 1.00 | 1.00 | **0.19** | 1.00 |
| CDR1788 |  | hypothetical protein | 1.00 | 1.00 | 1.00 | 1.00 | **2.21** |
| CDR1809 |  | Fragment of conserved hypothetical protein (N-terminal region) | **0.42** | **0.44** | 1.00 | 1.00 | 1.00 |
| CDR1810 |  | Conserved hypothetical protein | **0.11** | **0.14** | 1.00 | 1.00 | 1.00 |
| CDR1811 |  | Fragment of ABC-type transport system. substrate-binding protein (Part 1) | **0.14** | **0.15** | 1.00 | **3.61** | 1.00 |
| CDR1817 |  | Conserved hypothetical protein | 1.00 | 1.00 | 1.00 | **4.91** | **3.86** |
| CDR1818 |  | Putative phage-related cell wall hydrolase (endolysin) | 1.00 | 1.00 | 1.00 | **4.97** | **3.28** |
| CDR1820 |  | Conserved hypothetical protein | **0.32** | 1.00 | 1.00 | 1.00 | **0.39** |
| CDR1824 |  | ABC-type transport system. permease | **0.30** | **0.31** | 1.00 | **7.17** | **4.59** |
| CDR1825 |  | Fragment of ABC transporter atp-binding protein | 1.00 | **0.46** | 1.00 | **5.01** | **4.07** |
| CDR1847 |  | transcriptional regulator | 1.00 | 1.00 | 1.00 | 1.00 | **3.00** |
| CDR1851 |  | Putative membrane protein | **0.12** | **0.10** | 1.00 | **0.31** | **0.14** |
| CDR1852 |  | Putative membrane protein | **0.33** | **0.24** | 1.00 | **0.29** | **0.26** |
| CDR1853 |  | Putative lipoprotein | **0.36** | **0.27** | 1.00 | **0.39** | **0.31** |
| CDR1858 | *spoVS* | Stage V sporulation protein S | **0.20** | 1.00 | 1.00 | 1.00 | **0.34** |
| CDR1863 |  | Putative membrane protein | **0.26** | **0.28** | 1.00 | **0.48** | **0.35** |
| CDR1864 |  | Conserved hypothetical protein | **0.17** | **0.28** | 1.00 | 1.00 | **0.24** |
| CDR1874 |  | putative Acyl-CoA N-acyltransferase | **0.39** | **0.38** | 1.00 | **3.28** | 1.00 |
| CDR1890 |  | Conserved hypothetical protein | **0.22** | 1.00 | 1.00 | 1.00 | **0.37** |
| CDR1917 |  | Conserved hypothetical protein | **0.17** | 1.00 | 1.00 | 1.00 | **0.54** |
| CDR1925 | *fldX* | Flavodoxin | 1.00 | 1.00 | 1.00 | **0.38** | **0.33** |
| CDR1926 | *isp* | Intracellular serine protease | **0.29** | **0.33** | 1.00 | 1.00 | **0.27** |
| CDR1927 | *ilvD* | Dihydroxy-acid dehydratase | **0.35** | 1.00 | 1.00 | 1.00 | **0.42** |
| CDR1929 |  | Conserved hypothetical protein | 1.00 | 1.00 | 1.00 | 1.00 | **2.61** |
| CDR1940 |  | N-carbamoyl-L-amino acid hydrolase | 1.00 | 1.00 | 1.00 | 1.00 | **0.45** |
| CDR1954 |  | Conserved hypothetical protein | 1.00 | 1.00 | 1.00 | **0.42** | 1.00 |
| CDR1962 |  | Conserved hypothetical protein | 1.00 | 1.00 | 1.00 | **6.77** | **4.74** |
| CDR1967 |  | Putative lipid kinase | **0.26** | **0.34** | 1.00 | 1.00 | **0.44** |
| CDR1968 |  | Conserved hypothetical protein. DUF1121 family | **0.42** | 1.00 | 1.00 | 1.00 | **0.52** |
| CDR1969 |  | Conserved hypothetical protein | **0.37** | 1.00 | 1.00 | 1.00 | **0.43** |
| CDR1970 |  | Conserved hypothetical protein | 1.00 | 1.00 | 1.00 | 1.00 | **0.38** |
| CDR1983 |  | Conserved hypothetical protein | 1.00 | 1.00 | 1.00 | 1.00 | **0.47** |
| CDR2005 |  | Conserved hypothetical protein | **0.32** | **0.43** | 1.00 | 1.00 | **0.43** |
| CDR2014 |  | Xanthine/uracil/thiamine/ascorbate permease family protein | 1.00 | 1.00 | 1.00 | **0.49** | **2.57** |
| CDR2019 |  | Conserved hypothetical protein | **0.35** | **0.27** | 1.00 | 1.00 | 1.00 |
| CDR2023 | *bipA* | GTP-binding protein BipA | **2.05** | 1.00 | 1.00 | 1.00 | **2.47** |
| CDR2027 |  | Putative 2-nitropropane dioxygenase | **2.27** | 1.00 | 1.00 | 1.00 | **2.31** |
| CDR2028 |  | Conserved hypothetical protein | **0.09** | **0.09** | 1.00 | **0.23** | **0.21** |
| CDR2063 |  | Conserved hypothetical protein | **0.23** | 1.00 | 1.00 | 1.00 | **0.39** |
| CDR2064 | *gabT* | 4-aminobutyrate aminotransferase | 1.00 | 1.00 | 1.00 | 1.00 | **0.37** |
| CDR2072 | *msrAB* | Peptide methionine sulfoxide reductase MsrA/MsrB | 1.00 | **0.35** | 1.00 | 1.00 | 1.00 |
| CDR2074 | *hcp* | Hydroxylamine reductase | 1.00 | 1.00 | 1.00 | 1.00 | **3.03** |
| CDR2077 |  | Fragment of putative sodium:dicarboxylate symporter (Part 1) | 1.00 | 1.00 | 1.00 | 1.00 | **3.67** |
| CDR2080 |  | ABC-type transport system. cystine/aminoacid-family extracellular solute-binding protein | 1.00 | 1.00 | 1.00 | **2.59** | **2.55** |
| CDR2081 |  | ABC-type transport system. cystine/aminoacid-family permease | 1.00 | 1.00 | 1.00 | **2.32** | **2.44** |
| CDR2082 |  | ABC-type transport system. cystine/aminoacid-family permease | **2.93** | 1.00 | 1.00 | **2.91** | **3.08** |
| CDR2083 |  | ABC-type transport system. cystine/aminoacid-family extracellular solute-binding protein | 1.00 | 1.00 | 1.00 | **2.40** | **2.38** |
| CDR2089 |  | Putative N-acetylmuramoyl-L-alanine amidase | **0.31** | **0.36** | 1.00 | **6.47** | **4.62** |
| CDR2090 |  | Conserved hypothetical protein | **0.54** | 1.00 | 1.00 | 1.00 | **0.46** |
| CDR2128 |  | Putative membrane protein | 1.00 | **0.43** | 1.00 | 1.00 | 1.00 |
| CDR2145 |  | Conserved hypothetical protein | **0.07** | **0.08** | 1.00 | **0.33** | **0.14** |
| CDR2146 | *cspC* | Germination- specific protease | **0.11** | **0.13** | 1.00 | **0.29** | **0.14** |
| CDR2147 | *cspBA* | Subtilisin-like serine germination related protease | **0.10** | **0.11** | 1.00 | **0.23** | **0.12** |
| CDR2151 |  | Conserved hypothetical protein | **0.19** | **0.19** | 1.00 | **0.29** | **0.38** |
| CDR2160 |  | Putative peptidase. M24 family | **0.38** | **0.39** | 1.00 | **1.94** | 1.00 |
| CDR2161 |  | Transporter. Major Facilitator Superfamily (MFS) | **0.24** | **0.24** | 1.00 | 1.00 | **0.42** |
| CDR2175 |  | Class II aldolase | **0.21** | **0.29** | 1.00 | 1.00 | **2.56** |
| CDR2176 | *araD* | L-ribulose-5-phosphate 4-epimerase (Phosphoribulose isomerase) | 1.00 | **0.49** | 1.00 | 1.00 | **2.45** |
| CDR2178 |  | PTS system. fructose/mannitol family IIC component | **0.44** | **0.50** | 1.00 | 1.00 | 1.00 |
| CDR2179 |  | PTS system. fructose/mannitol family IIB component | **0.38** | 1.00 | 1.00 | 1.00 | **2.37** |
| CDR2181 |  | Sigma-54-dependent regulator. PTS regulatory domain | **0.21** | **0.27** | 1.00 | 1.00 | 1.00 |
| CDR2207 |  | Putative phosphohexomutase | 1.00 | **2.70** | 1.00 | 1.00 | **2.34** |
| CDR2210 | *tkt'* | Transketolase. central and C-terminal (Sedoheptulose-7-phosphate:D-glyceraldehyde-3-phosphate glycolaldehydetransferase) | **2.20** | **2.50** | 1.00 | 1.00 | **3.61** |
| CDR2211 | *tkt* | Transketolase. N-terminal (Sedoheptulose-7-phosphate:D-glyceraldehyde-3-phosphate glycolaldehydetransferase) | **3.02** | **3.44** | 1.00 | 1.00 | **3.14** |
| CDR2212 |  | Putative sugar-phosphate dehydrogenase | **5.73** | **11.45** | 1.00 | **3.43** | **6.78** |
| CDR2213 |  | Putative sugar-phosphate dehydrogenase | **5.57** | **12.97** | 1.00 | **4.91** | **7.79** |
| CDR2214 | *gatC* | PTS system. galactitol-specific IIC component | **8.57** | **9.70** | 1.00 | **7.68** | **6.74** |
| CDR2215 |  | PTS system. lactose/cellobiose specific IIB component | **6.13** | **13.98** | 1.00 | **3.29** | **7.25** |
| CDR2216 | *gatA* | PTS system. galactitol-specific IIA component | 1.00 | **6.31** | 1.00 | 1.00 | **3.71** |
| CDR2227 | *4hbD* | 4-hydroxybutyrate dehydrogenase (4-hydroxybutanoate:NAD+ oxidoreductase) | **0.33** | 1.00 | 1.00 | 1.00 | **0.34** |
| CDR2228 | *cat2* | 4-hydroxybutyrate CoA transferase | **0.42** | 1.00 | 1.00 | 1.00 | **0.33** |
| CDR2229 |  | Conserved hypothetical protein | **0.42** | 1.00 | 1.00 | 1.00 | **0.35** |
| CDR2230 | *abfD* | Gamma-aminobutyrate metabolism dehydratase/isomerase [includes: 4- hydroxybutyryl-coa dehydratase vinylacetyl-coa-delta-isomerase] | **0.24** | 1.00 | 1.00 | 1.00 | **0.24** |
| CDR2231 | *sucD* | Succinate-semialdehyde dehydrogenase (NAD(P)+) | **0.45** | 1.00 | 1.00 | 1.00 | **0.31** |
| CDR2232 | *cat1* | Succinyl-CoA:coenzyme A transferase | **0.40** | 1.00 | 1.00 | 1.00 | **0.40** |
| CDR2235 |  | Putative membrane protein | 1.00 | 1.00 | 1.00 | **5.07** | **3.52** |
| CDR2247 |  | Conserved hypothetical protein | 1.00 | 1.00 | 1.00 | 1.00 | **2.05** |
| CDR2248 |  | ABC-type transport system. nitrate/sulfonate/taurine ATP-binding protein | 1.00 | 1.00 | 1.00 | 1.00 | **2.14** |
| CDR2249 |  | Fragment of ABC-type transport system. permease (Part 1) | 1.00 | 1.00 | 1.00 | **2.15** | **2.16** |
| CDR2250 |  | Conserved hypothetical protein | 1.00 | 1.00 | 1.00 | **2.99** | **2.40** |
| CDR2252 |  | ABC-type transport system. nitrate/sulfonate/taurine extracellular solute-binding protein | 1.00 | 1.00 | 1.00 | 1.00 | **2.16** |
| CDR2253 |  | Conserved hypothetical protein | 1.00 | 1.00 | 1.00 | **2.36** | **2.67** |
| CDR2254 |  | ABC-type transport system. multidrug-family permease | 1.00 | 1.00 | 1.00 | 1.00 | **2.25** |
| CDR2255 |  | Conserved hypothetical protein | 1.00 | **2.17** | 1.00 | **2.55** | **2.05** |
| CDR2256 |  | Conserved hypothetical protein | 1.00 | 1.00 | 1.00 | **2.31** | 1.00 |
| CDR2260 |  | Putative CstA-like carbon starvation protein | **0.14** | 1.00 | 1.00 | 1.00 | **0.30** |
| CDR2261 |  | Conserved hypothetical protein | **0.11** | **0.16** | 1.00 | **0.33** | **0.14** |
| CDR2262 |  | Conserved hypothetical protein | **0.03** | **0.06** | 1.00 | **0.39** | **0.12** |
| CDR2266 | *buk* | Butyrate kinase (Branched-chain carboxylic acid kinase) (BK) | **0.19** | 1.00 | 1.00 | 1.00 | **0.26** |
| CDR2267 | *iorB* | Indole pyruvate ferredoxin/flavodoxin oxidoreductase | **0.18** | 1.00 | 1.00 | 1.00 | **0.24** |
| CDR2268 | *iorA* | Indole pyruvate ferredoxin/flavodoxin oxidoreductase | **0.19** | 1.00 | 1.00 | 1.00 | **0.24** |
| CDR2269 |  | Putative pyridoxal phosphate-dependent transferase | **0.22** | 1.00 | 1.00 | 1.00 | **0.25** |
| CDR2274 |  | Alpha-hydroxy acid dehydrogenase. FMN-dependent | **0.25** | 1.00 | 1.00 | **0.45** | **0.34** |
| CDR2289 |  | Conserved hypothetical protein | **0.21** | **0.20** | 1.00 | **6.70** | 1.00 |
| CDR2290 | *cotJB2* | Spore coat peptide assembly protein CotJB 2 | **0.16** | **0.15** | 1.00 | **5.98** | 1.00 |
| CDR2291 | *cotD* | Spore coat protein CotD manganese catalase | **0.16** | **0.16** | 1.00 | **6.89** | **3.91** |
| CDR2300 |  | Conserved hypothetical protein | **0.23** | **0.24** | 1.00 | **4.83** | 1.00 |
| CDR2312 |  | Putative amidohydrolase. M20D family | 1.00 | **6.64** | 1.00 | 1.00 | 1.00 |
| CDR2313 |  | Sodium:alanine symporter | 1.00 | **6.10** | 1.00 | 1.00 | 1.00 |
| CDR2314 |  | Transporter. Major Facilitator Superfamily (MFS) | **0.31** | **5.98** | 1.00 | **0.33** | **0.28** |
| CDR2315 |  | Putative pyridoxal phosphate-dependent transferase | 1.00 | **3.89** | 1.00 | 1.00 | 1.00 |
| CDR2316 | *ptb* | Phosphate butyryltransferase (Phosphotransbutyrylase) | 1.00 | **4.93** | 1.00 | 1.00 | 1.00 |
| CDR2317 | *buk1* | Butyrate kinase 1 (BK 1) (Branched-chain carboxylic acid kinase 1) | **0.49** | **6.05** | 1.00 | 1.00 | **0.49** |
| CDR2318 |  | Putative flavodoxin/ferredoxin oxidoreductase gamma subunit | **0.27** | 1.00 | 1.00 | **0.34** | **0.29** |
| CDR2319 |  | Putative flavodoxin/ferredoxin oxidoreductase beta subunit | **0.41** | 1.00 | 1.00 | 1.00 | **0.42** |
| CDR2320 |  | Putative flavodoxin/ferredoxin oxidoreductase alpha subunit | **0.28** | 1.00 | 1.00 | 1.00 | **0.34** |
| CDR2326 |  | Conserved hypothetical protein | **0.33** | 1.00 | 1.00 | 1.00 | **0.36** |
| CDR2327 | *recO* | DNA repair protein RecO (Recombination protein O) | 1.00 | 1.00 | 1.00 | 1.00 | **0.41** |
| CDR2328 |  | Putative divalent cation transporter. MgtE family | **0.33** | **0.36** | 1.00 | 1.00 | **0.51** |
| CDR2330 | *cdd* | Cytidine deaminase (Cytidine aminohydrolase) (CDA) | 1.00 | 1.00 | 1.00 | 1.00 | **0.52** |
| CDR2332 |  | Putative metal-dependent hydrolase | **0.33** | **0.29** | 1.00 | **0.40** | **0.30** |
| CDR2333 |  | PhoH-like protein | **0.35** | **0.40** | 1.00 | **0.47** | **0.45** |
| CDR2334 | *spoIV* | Stage IV sporulation protein | **0.28** | **0.29** | 1.00 | 1.00 | **0.35** |
| CDR2335 |  | Conserved hypothetical protein | **0.08** | **0.09** | 1.00 | 1.00 | **0.18** |
| CDR2336 |  | Putative ribosome-associated sigma 54 modulation protein | **0.23** | **0.20** | 1.00 | **0.39** | **0.24** |
| CDR2337 |  | Putative transmembrane signaling protein. TspO/MBR family | **0.18** | **0.18** | 1.00 | **0.30** | **0.17** |
| CDR2353 | *dnaJ* | Chaperone protein DnaJ | 1.00 | 1.00 | 1.00 | 1.00 | **2.67** |
| CDR2354 | *dnaK* | Chaperone protein dnaK (Heat shock protein 70) (Heat shock 70 kDa protein) (HSP70) | 1.00 | 1.00 | 1.00 | 1.00 | **2.83** |
| CDR2355 | *grpE* | Protein grpE (HSP-70 cofactor) | 1.00 | 1.00 | 1.00 | 1.00 | **2.81** |
| CDR2356 | *hrcA* | Transcriptional regulator. Heat-inducible repressor HrcA | 1.00 | 1.00 | 1.00 | 1.00 | **2.08** |
| CDR2361 |  | Conserved hypothetical protein | **0.08** | **0.08** | 1.00 | **0.09** | **0.10** |
| CDR2362 | *spoIIP* | Stage II sporulation protein P | **0.25** | **0.23** | 1.00 | **0.37** | **0.24** |
| CDR2363 | *gpr* | Spore endopeptidase | **0.22** | **0.26** | 1.00 | **0.36** | **0.21** |
| CDR2366 | *rpsT* | 30S ribosomal protein S20 | 1.00 | 1.00 | 1.00 | 1.00 | **2.26** |
| CDR2391 |  | Conserved hypothetical protein | 1.00 | 1.00 | 1.00 | **0.36** | **0.31** |
| CDR2399 |  | Conserved hypothetical protein | 1.00 | 1.00 | 1.00 | **2.21** | **5.19** |
| CDR2400 |  | Transcriptional regulator. AraC family | **2.70** | 1.00 | 1.00 | **2.03** | **4.30** |
| CDR2401 |  | Putative glycoside hydrolase. family 4 | 1.00 | 1.00 | 1.00 | **2.59** | **6.07** |
| CDR2402 |  | PTS system. glucose-like IIBC component | 1.00 | 1.00 | 1.00 | **2.21** | **7.08** |
| CDR2403 |  | Transcription antiterminator. PTS operon regulator | 1.00 | 1.00 | 1.00 | 1.00 | **4.89** |
| CDR2404 |  | PTS system. glucose-like IIA component | 1.00 | 1.00 | 1.00 | **2.54** | **7.40** |
| CDR2434 |  | Conserved hypothetical protein | 1.00 | 1.00 | 1.00 | 1.00 | **2.89** |
| CDR2435 |  | ABC-type transport system. sugar-family permease | **2.89** | 1.00 | 1.00 | 1.00 | **2.95** |
| CDR2436 |  | ABC-type transport system. sugar-family permease | **2.79** | 1.00 | 1.00 | 1.00 | **3.31** |
| CDR2437 |  | ABC-type transport system. sugar-family extracellular solute-binding protein | 1.00 | 1.00 | 1.00 | 1.00 | **2.67** |
| CDR2448 | *rpmB* | 50S ribosomal protein L28 | 1.00 | 1.00 | 1.00 | 1.00 | **2.00** |
| CDR2487 | *cstA* | Carbon starvation protein. CstA | **2.80** | 1.00 | 1.00 | 1.00 | **3.20** |
| CDR2492 |  | Fragment of ADP-ribosyltransferase CdtAB (Part 7) | **0.27** | **0.29** | 1.00 | 1.00 | 1.00 |
| CDR2503 |  | Putative cell-division initiation protein DivIVA | **0.42** | 1.00 | 1.00 | 1.00 | **0.50** |
| CDR2504 |  | Putative RNA-binding cell division protein | 1.00 | 1.00 | 1.00 | 1.00 | **0.55** |
| CDR2505 |  | Conserved hypothetical protein | **0.36** | 1.00 | 1.00 | 1.00 | **0.49** |
| CDR2506 | *sepF* | Cell division protein SepF | **0.46** | 1.00 | 1.00 | 1.00 | **0.48** |
| CDR2508 |  | Conserved hypothetical protein | **0.30** | 1.00 | 1.00 | 1.00 | **0.37** |
| CDR2509 |  | Putative membrane protein | **0.19** | 1.00 | 1.00 | 1.00 | **0.31** |
| CDR2510 |  | Conserved hypothetical protein | **2.57** | **2.71** | 1.00 | 1.00 | **2.34** |
| CDR2511 |  | Conserved hypothetical protein | **3.69** | **2.12** | 1.00 | 1.00 | 1.00 |
| CDR2513 | *spoIVA* | Stage IV sporulation protein A | **0.04** | **0.04** | 1.00 | **0.31** | **0.09** |
| CDR2525 |  | Two-component sensor histidine kinase | **0.24** | **0.24** | 1.00 | **0.36** | **0.24** |
| CDR2526 |  | Two-component response regulator | **0.41** | **0.41** | 1.00 | **0.46** | **0.40** |
| CDR2527 |  | Putative laccase protein | **0.19** | **0.17** | 1.00 | **0.29** | **0.22** |
| CDR2528 | *nrdR* | Transcriptional regulator. repressor NrdR family | **0.25** | **0.25** | 1.00 | **0.35** | **0.31** |
| CDR2529 |  | Putative sporulation protein | **0.25** | **0.23** | 1.00 | **0.31** | **0.31** |
| CDR2530 | *sigG* | RNA polymerase sigma-G factor | **0.06** | **0.09** | 1.00 | **0.25** | **0.10** |
| CDR2531 | *sigE* | RNA polymerase sigma-E factor | **0.13** | **0.19** | 1.00 | **0.25** | **0.17** |
| CDR2532 | *spoIIGA* | Sporulation sigma-E factor processing peptidase | **0.29** | **0.34** | 1.00 | **0.34** | **0.29** |
| CDR2533 |  | ABC-type transport system. sugar-family extracellular solute-binding protein | 1.00 | 1.00 | 1.00 | 1.00 | **2.94** |
| CDR2539 | *murG* | UDP-N-acetylglucosamine--N-acetylmuramyl-(pentapeptide) pyrophosphoryl-undecaprenol N-acetylglucosamine transferase | **0.37** | **0.46** | 1.00 | 1.00 | **0.45** |
| CDR2540 | *spoVE* | Cell division/stage V sporulation protein | **0.32** | **0.47** | 1.00 | 1.00 | **0.39** |
| CDR2541 | *murD* | UDP-N-acetylmuramoylalanine--D-glutamate ligase (UDP-N- acetylmuramoyl-L-alanyl-D-glutamate synthetase) (D-glutamic acid- adding enzyme) | **0.42** | 1.00 | 1.00 | 1.00 | **0.42** |
| CDR2544 | *spoVD* | Stage V sporulation protein D (Sporulation-specific penicillin-binding protein) | **0.28** | **0.35** | 1.00 | 1.00 | **0.29** |
| CDR2545 |  | Putative lipoprotein | **0.23** | **0.32** | 1.00 | **0.41** | **0.25** |
| CDR2548 |  | Putative peptidase. M16 family | **0.23** | **0.22** | 1.00 | **0.29** | **0.28** |
| CDR2549 |  | Putative peptidase. M16 family | **0.39** | **0.36** | 1.00 | **0.47** | **0.41** |
| CDR2557 |  | Putative Na(+)/H(+) antiporter | 1.00 | 1.00 | 1.00 | **2.20** | **2.58** |
| CDR2558 | *appF* | ABC-type transport system. ATP-binding protein | 1.00 | 1.00 | 1.00 | **2.46** | **3.02** |
| CDR2559 | *appD* | ABC-type transport system. ATP-binding protein | 1.00 | 1.00 | 1.00 | 1.00 | **2.51** |
| CDR2560 | *appA* | ABC-type transport system. oligopeptide-family solute-binding protein | 1.00 | 1.00 | 1.00 | **3.34** | **5.00** |
| CDR2561 | *appB* | ABC-type transport system. oligopeptide-family permease protein | **2.45** | 1.00 | 1.00 | **2.96** | **3.78** |
| CDR2562 | *appC* | ABC-type transport system. oligopeptide-family permease protein | **1.92** | 1.00 | 1.00 | **2.19** | **2.45** |
| CDR2564 | *thlA2* | Acetoacetyl-CoA thiolase 2 | 1.00 | 1.00 | 1.00 | 1.00 | **0.41** |
| CDR2565 | *scoA* | Succinyl CoA:3-oxoacid CoA-transferase subunit A | 1.00 | 1.00 | 1.00 | 1.00 | **0.45** |
| CDR2567 | *bdhA* | 3-hydroxybutyrate dehydrogenase | 1.00 | 1.00 | 1.00 | 1.00 | **0.41** |
| CDR2568 |  | Putative permease | 1.00 | 1.00 | 1.00 | 1.00 | **0.42** |
| CDR2575 |  | Conserved hypothetical protein | **0.36** | **0.43** | 1.00 | 1.00 | 1.00 |
| CDR2576 | *sspA* | Small. acid-soluble spore protein alpha | **0.02** | **0.01** | 1.00 | 1.00 | 1.00 |
| CDR2580 |  | Putative threonine-phosphate decarboxylase | 1.00 | **0.49** | 1.00 | 1.00 | 1.00 |
| CDR2581 |  | Sodium:dicarboxylate symporter family protein | **0.30** | **0.34** | 1.00 | 1.00 | 1.00 |
| CDR2592 |  | Putative permease | 1.00 | 1.00 | 1.00 | 1.00 | **2.19** |
| CDR2613 |  | Putative polysaccharide deacetylase | 1.00 | 1.00 | 1.00 | 1.00 | **0.33** |
| CDR2614 |  | Putative monogalactosyldiacylglycerol synthase | 1.00 | 1.00 | 1.00 | 1.00 | **0.31** |
| CDR2615 | *glyA* | Serine hydroxymethyltransferase | **0.36** | 1.00 | 1.00 | 1.00 | **0.34** |
| CDR2626 |  | Putative nitrilase/cyanide hydratase and apolipoprotein N-acyltransferase | **3.25** | 1.00 | 1.00 | **6.24** | **2.92** |
| CDR2627 |  | Putative cytosine permease | 1.00 | 1.00 | 1.00 | **4.22** | **2.41** |
| CDR2642 |  | Conserved hypothetical protein | 1.00 | 1.00 | 1.00 | **0.36** | **0.19** |
| CDR2649 |  | Putative N-acetylmuramoyl-L-alanine amidase | **0.45** | **0.39** | 1.00 | 1.00 | **0.47** |
| CDR2650 | *uppS* | Putative undecaprenyl pyrophosphate synthetase | **0.23** | **0.24** | 1.00 | **0.36** | **0.31** |
| CDR2654 |  | Transcriptional regulator. LytR family | **2.86** | 1.00 | 1.00 | 1.00 | **2.32** |
| CDR2685 | *cwp10* | Cell surface protein | 1.00 | 1.00 | 1.00 | 1.00 | **2.33** |
| CDR2686 |  | Putative calcium-binding adhesion protein | **2.33** | 1.00 | 1.00 | 1.00 | **2.27** |
| CDR2689 |  | Putative membrane protein | **0.09** | **0.10** | 1.00 | **0.29** | **0.11** |
| CDR2697 |  | Conserved hypothetical protein | **0.06** | **0.06** | 1.00 | **0.13** | **0.07** |
| CDR2698 |  | Conserved hypothetical protein. DUF1540 family | **0.16** | **0.06** | 1.00 | **0.37** | **0.10** |
| CDR2719 |  | Putative pyridoxal phosphate-dependent transferase | **0.40** | **0.30** | 1.00 | **0.37** | **0.39** |
| CDR2724 |  | Putative calcium-transporting ATPase | **0.15** | **0.17** | 1.00 | **0.23** | **0.16** |
| CDR2736 | *rbr* | Rubrerythrin | **0.16** | **0.18** | 1.00 | 1.00 | **0.23** |
| CDR2747 |  | Putative membrane protein | **0.46** | **0.46** | 1.00 | 1.00 | **0.43** |
| CDR2755 |  | Putative hydrolase | **0.11** | **0.11** | 1.00 | **0.30** | **0.14** |
| CDR2756 |  | Putative bacterioferritin | **0.26** | **0.25** | 1.00 | **0.32** | **0.24** |
| CDR2787 | *atpD* | V-type ATP synthase subunit D | **0.23** | 1.00 | 1.00 | 1.00 | **0.25** |
| CDR2788 | *atpB* | V-type ATP synthase beta chain (V-type ATPase subunit B) | **0.31** | 1.00 | 1.00 | 1.00 | **0.24** |
| CDR2789 | *atpA* | V-type ATP synthase alpha chain (V-type ATPase subunit A) | **0.24** | 1.00 | 1.00 | 1.00 | **0.23** |
| CDR2790 | *atpF* | V-type ATP synthase subunit F | **0.31** | 1.00 | 1.00 | 1.00 | **0.28** |
| CDR2791 | *atpC* | V-type ATP synthase subunit C | 1.00 | 1.00 | 1.00 | 1.00 | **0.24** |
| CDR2792 | *atpE* | V-type ATP synthase subunit E (V-type ATPase subunit E) | 1.00 | 1.00 | 1.00 | 1.00 | **0.22** |
| CDR2793 | *atpK* | V-type ATP synthase subunit K | **0.25** | 1.00 | 1.00 | 1.00 | **0.24** |
| CDR2794 | *atpI* | V-type sodium ATP synthase subunit I | **0.23** | 1.00 | 1.00 | 1.00 | **0.24** |
| CDR2795 |  | Conserved hypothetical protein | **0.30** | 1.00 | 1.00 | 1.00 | **0.32** |
| CDR2796 |  | Conserved hypothetical protein | **0.51** | 1.00 | 1.00 | 1.00 | **0.49** |
| CDR2800 | *adhE* | Aldehyde-alcohol dehydrogenase | 1.00 | **0.48** | 1.00 | 1.00 | 1.00 |
| CDR2802 | *spoVFB* | Dipicolinate synthase subunit B | **0.33** | **0.33** | 1.00 | **2.50** | 1.00 |
| CDR2803 | *dpaA* | Dipicolinate synthase subunit A | **0.28** | **0.29** | 1.00 | **2.59** | 1.00 |
| CDR2828 |  | Conserved hypothetical protein | **3.35** | **2.73** | 1.00 | 1.00 | 1.00 |
| CDR2839 |  | Putative N-terminus region of carbohydrate hydrolase. SAF domain | **5.01** | 1.00 | 1.00 | **2.40** | **1.83** |
| CDR2840 | *kdgT2* | 2-keto-3-deoxygluconate permease 2 | 1.00 | 1.00 | 1.00 | **2.20** | 1.00 |
| CDR2842 |  | Alcohol dehydrogenase | **5.80** | 1.00 | 1.00 | **1.97** | 1.00 |
| CDR2854 |  | Putative redox-active protein | 1.00 | 1.00 | 1.00 | **2.43** | **2.45** |
| CDR2855 |  | Putative transporter | 1.00 | 1.00 | 1.00 | 1.00 | **2.30** |
| CDR2862 |  | PTS system. glucose-like IIA component | 1.00 | 1.00 | 1.00 | **2.40** | **3.12** |
| CDR2865 |  | PTS system. glucose-like IIBC component | 1.00 | 1.00 | 1.00 | **2.76** | 1.00 |
| CDR2866 |  | Transcription antiterminator. PTS operon regulator | 1.00 | 1.00 | 1.00 | **2.04** | 1.00 |
| CDR2867 |  | Putative pyridoxal phosphate-dependent transferase | 1.00 | 1.00 | 1.00 | **4.30** | **3.16** |
| CDR2871 |  | Transporter. Major Facilitator Superfamily (MFS) | **2.33** | 1.00 | 1.00 | 1.00 | **2.96** |
| CDR2872 |  | Transcriptional regulator. CarD family | **0.44** | 1.00 | 1.00 | 1.00 | **0.41** |
| CDR2907 |  | Putative membrane protein | 1.00 | 1.00 | 1.00 | **2.82** | 1.00 |
| CDR2937 |  | Putative amidohydrolase. M20D family | **3.01** | 1.00 | 1.00 | 1.00 | **2.45** |
| CDR2978 |  | PTS system. fructose-like IIABC component | **8.65** | 1.00 | 1.00 | **2.85** | 1.00 |
| CDR3026 | *eno* | Enolase (2-phosphoglycerate dehydratase) (2-phospho-D-glycerate hydro-lyase) | 1.00 | 1.00 | 1.00 | 1.00 | **2.06** |
| CDR3028 | *tpi* | Triosephosphate isomerase (TIM) (Triose-phosphate isomerase) | 1.00 | 1.00 | 1.00 | 1.00 | **1.91** |
| CDR3029 | *pgk* | Phosphoglycerate kinase | 1.00 | 1.00 | 1.00 | 1.00 | **2.20** |
| CDR3030 | *gapA* | Glyceraldehyde-3-phosphate dehydrogenase (GAPDH) | 1.00 | 1.00 | 1.00 | 1.00 | **2.10** |
| CDR3033 |  | Putative xanthine dehydrogenase | **0.36** | **0.39** | 1.00 | 1.00 | **0.28** |
| CDR3034 |  | D-hydantoinase (Dihydropyrimidinase) | **0.37** | 1.00 | 1.00 | **0.37** | **0.22** |
| CDR3035 | *pyrD* | Dihydroorotate dehydrogenase. catalytic subunit | **0.40** | 1.00 | 1.00 | 1.00 | **0.29** |
| CDR3036 |  | Putative purine permease | **0.40** | 1.00 | 1.00 | 1.00 | **0.28** |
| CDR3037 |  | Putative amidohydrolase | 1.00 | 1.00 | 1.00 | **0.40** | **0.31** |
| CDR3038 |  | Putative D-aminoacylase | 1.00 | 1.00 | 1.00 | **0.37** | **0.27** |
| CDR3039 |  | Putative peptidase. M20 family | **0.40** | 1.00 | 1.00 | **0.36** | **0.29** |
| CDR3040 | *dpaL* | Diaminopropionate ammonia-lyase | 1.00 | 1.00 | 1.00 | 1.00 | **0.40** |
| CDR3043 | *tdcF* | Putative regulatory endoribonuclease | 1.00 | **3.29** | 1.00 | 1.00 | **0.53** |
| CDR3044 |  | Conserved hypothetical protein | 1.00 | 1.00 | 1.00 | **2.86** | 1.00 |
| CDR3054 |  | Transcriptional regulator. AraC family | 1.00 | 1.00 | 1.00 | 1.00 | **3.08** |
| CDR3090 | *bclA2* | Exosporium glycoprotein | 1.00 | 1.00 | 1.00 | **5.96** | **3.15** |
| CDR3094 |  | Conserved hypothetical protein | **0.16** | **0.14** | 1.00 | **0.27** | **0.18** |
| CDR3095 | *ssb* | Single-stranded DNA-binding protein | **0.08** | **0.08** | 1.00 | **0.31** | **0.12** |
| CDR3096 |  | Putative membrane protein | 1.00 | **0.30** | 1.00 | 1.00 | 1.00 |
| CDR3099 | *prdE* | Proline reductase PrdE | 1.00 | **0.44** | 1.00 | 1.00 | 1.00 |
| CDR3101 | *prdB* | Proline reductase (selenocysteine) | 1.00 | **0.33** | 1.00 | 1.00 | 1.00 |
| CDR3102 |  | Conserved hypothetical protein | 1.00 | **0.44** | 1.00 | 1.00 | 1.00 |
| CDR3103 | *prdA* | D-proline reductase proprotein prdA | 1.00 | **0.37** | 1.00 | 1.00 | 1.00 |
| CDR3106 |  | Polysaccharide deacetylase | **0.16** | **0.16** | 1.00 | **0.38** | **0.18** |
| CDR3107 | *sspA* | Small. acid-soluble spore protein alpha | **0.04** | **0.03** | 1.00 | 1.00 | 1.00 |
| CDR3109 |  | Putative dehydrogenase | 1.00 | **0.35** | 1.00 | 1.00 | 1.00 |
| CDR3115 |  | Putative polysaccharide deacetylase | **0.28** | **0.27** | 1.00 | **0.44** | **0.27** |
| CDR3116 |  | Iron hydrogenase | **0.19** | **0.20** | 1.00 | 1.00 | **0.24** |
| CDR3136 |  | Putative phosphosugar isomerase | **0.37** | 1.00 | 1.00 | 1.00 | **3.51** |
| CDR3137 |  | PTS system. mannose/fructose/sorbose IID component | 1.00 | 1.00 | 1.00 | 1.00 | **2.43** |
| CDR3138 |  | PTS system. mannose/fructose/sorbose IIC component | **0.34** | 1.00 | 1.00 | 1.00 | **3.53** |
| CDR3139 |  | PTS system. mannose/fructose/sorbose IIA component | **0.40** | 1.00 | 1.00 | 1.00 | **4.04** |
| CDR3140 |  | PTS system. mannose/fructose/sorbose IIB component | 1.00 | 1.00 | 1.00 | 1.00 | **3.37** |
| CDR3143 | *pflD* | Pyruvate formate-lyase | **0.38** | **0.33** | 1.00 | 1.00 | **0.36** |
| CDR3144 | *pflE* | Pyruvate formate-lyase (activating enzyme) | 1.00 | 1.00 | 1.00 | 1.00 | **0.53** |
| CDR3158 |  | Conserved hypothetical protein | 1.00 | 1.00 | 1.00 | 1.00 | **0.41** |
| CDR3159 |  | Putative ATP/GTP-binding protein | **0.11** | **0.10** | 1.00 | 1.00 | **0.19** |
| CDR3193 | *bclA3* | Exosporium glycoprotein | **0.24** | **0.26** | 1.00 | **5.69** | 1.00 |
| CDR3194 | *sgtA* | Glycosyl transferase | **0.32** | **0.29** | 1.00 | 1.00 | 1.00 |
| CDR3228 |  | Putative iron-only hydrogenase.electron-transferring subunit HymB-like | 1.00 | 1.00 | 1.00 | 1.00 | **0.56** |
| CDR3229 |  | Putative iron-only hydrogenase. catalytic subunit HymC-like | **0.37** | 1.00 | 1.00 | 1.00 | **0.45** |
| CDR3263 |  | Putative pyridoxal phosphate-dependent transferase (PLP-dependent transferase) | 1.00 | **3.62** | 1.00 | 1.00 | 1.00 |
| CDR3264 |  | Putative peptidase. M24 family | 1.00 | **2.79** | 1.00 | 1.00 | 1.00 |
| CDR3265 |  | PTS system. lactose/cellobiose-family IIC component | 1.00 | **2.18** | 1.00 | 1.00 | 1.00 |
| CDR3266 |  | PTS system. lactose/cellobiose-family IIB component | 1.00 | **2.07** | 1.00 | 1.00 | 1.00 |
| CDR3267 |  | PTS system. lactose/cellobiose-family IIA component | 1.00 | **3.23** | 1.00 | 1.00 | 1.00 |
| CDR3291 |  | Putative carboxy-terminal protease | **0.16** | **0.25** | 1.00 | **0.29** | **0.31** |
| CDR3293 |  | Conserved hypothetical protein | **0.09** | **0.09** | 1.00 | **0.28** | **0.13** |
| CDR3294 |  | Putative membrane protein | 1.00 | 1.00 | 1.00 | 1.00 | **0.47** |
| CDR3297 | *EndoA* | Endoribonuclease toxin | **0.23** | **0.17** | 1.00 | **0.46** | **0.31** |
| CDR3298 |  | Putative antitoxin EndoAI | **0.25** | **0.23** | 1.00 | 1.00 | **0.26** |
| CDR3299 | *alr2* | Alanine racemase 2 | **0.25** | **0.23** | 1.00 | 1.00 | **0.29** |
| CDR3300 |  | Putative lipoprotein | **0.21** | **0.21** | 1.00 | 1.00 | **0.24** |
| CDR3301 |  | Conserved hypothetical protein | **0.45** | **0.43** | 1.00 | 1.00 | **0.48** |
| CDR3302 | *acpS* | Holo-[acyl-carrier-protein] synthase | **0.36** | **0.28** | 1.00 | **0.44** | **0.33** |
| CDR3313 |  | Putative dCMP deaminase | **2.10** | **2.05** | 1.00 | 1.00 | 1.00 |
| CDR3326 |  | Putative oligoendopeptidase F. peptidase M3B family | **0.13** | **0.20** | 1.00 | **0.48** | **0.20** |
| CDR3327 | *spoIIE* | Phosphoprotein phosphatase | **0.01** | **0.09** | 1.00 | **0.36** | **0.09** |
| CDR3329 |  | Septum formation initiation protein | **0.35** | **0.27** | 1.00 | 1.00 | **0.30** |
| CDR3330 |  | Putative membrane protein | **0.40** | **0.35** | 1.00 | 1.00 | **0.34** |
| CDR3331 |  | Putative spore protein | 1.00 | **0.42** | 1.00 | 1.00 | **0.41** |
| CDR3333 | *hbs* | DNA-binding protein. non-specific | **0.45** | **0.51** | 1.00 | 1.00 | 1.00 |
| CDR3336 | *spoVT* | Stage V sporulation protein T | **0.09** | **0.09** | 1.00 | **0.34** | **0.14** |
| CDR3353 | *spoVG* | Regulator required for spore cortex synthesis | **0.22** | 1.00 | 1.00 | 1.00 | **0.44** |
| CDR3358 |  | Putative peptidase T. M20B family | **0.20** | **0.30** | 1.00 | 1.00 | **0.33** |
| CDR3359 |  | Conserved hypothetical protein | **0.18** | **0.18** | 1.00 | 1.00 | **0.22** |
| CDR3376 | *spmB* | Spore maturation protein B | **0.25** | **0.37** | 1.00 | **0.31** | **0.35** |
| CDR3387 |  | Putative membrane protein | **0.14** | **0.15** | 1.00 | 1.00 | **0.19** |
| CDR3400 | *sleB* | Spore-cortex-lytic protein | **0.08** | **0.17** | 1.00 | **0.29** | **0.11** |
| CDR3401 | *spoIIR* | Pro-sigma(E) endopeptidase (stage II sporulation) | **0.35** | **0.31** | 1.00 | 1.00 | **0.36** |
| CDR3404 | *SipL* | SpoIVA-Interacting protein. coat localization | **0.05** | **0.05** | 1.00 | **0.36** | **0.10** |
| CDR3406 |  | Sporulation-specific protease | 1.00 | 1.00 | 1.00 | **2.73** | 1.00 |
| CDR3416 |  | Resolvase | 1.00 | 1.00 | 1.00 | 1.00 | **2.01** |
| CDR3417 |  | Conserved hypothetical protein | 1.00 | 1.00 | 1.00 | 1.00 | **2.23** |
| CDR3418 |  | Conserved hypothetical protein | **0.06** | **0.15** | 1.00 | 1.00 | 1.00 |
| CDR3433 |  | Aminopeptidase | **0.36** | **0.50** | 1.00 | 1.00 | **0.40** |
| CDR3449 |  | Conserved hypothetical protein | 1.00 | 1.00 | 1.00 | **0.43** | **0.45** |
| CDR3450 |  | multidrug resistance protein | 1.00 | **0.45** | 1.00 | 1.00 | 1.00 |
| CDR3451 |  | Conserved hypothetical protein | **0.26** | **0.25** | 1.00 | **4.21** | 1.00 |
| CDR3482 |  | Conserved hypothetical protein | 1.00 | 1.00 | 1.00 | **37.17** | **29.33** |
| CDR3487 |  | Putative 4-hydroxy-2-oxoglutarate aldolase | 1.00 | **3.67** | 1.00 | 1.00 | 1.00 |
| CDR3488 | *dapA4* | Dihydrodipicolinate synthase 4 (DHDPS) | 1.00 | **2.49** | 1.00 | 1.00 | 1.00 |
| CDR3489 |  | Putative phosphosugar isomerase | 1.00 | **4.58** | 1.00 | 1.00 | 1.00 |
| CDR3491 |  | PTS system. fructose/mannitol-family IIB component | 1.00 | **3.49** | 1.00 | 1.00 | 1.00 |
| CDR3499 |  | Putative ribokinase family sugar kinase | 1.00 | **2.92** | 1.00 | 1.00 | 1.00 |
| CDR3506 | *licA* | PTS system. lichenan-specific IIA component | 1.00 | **3.66** | 1.00 | 1.00 | 1.00 |
| CDR3511 |  | Putative peptidase. M1 family | **0.13** | **0.15** | 1.00 | **0.21** | **0.15** |
| CDR3521 | *rpsR* | 30S ribosomal protein S18 | 1.00 | 1.00 | 1.00 | 1.00 | **2.07** |
| CDR3522 | *ssb* | Single-stranded DNA-binding protein (Helix-destabilizing protein) | 1.00 | 1.00 | 1.00 | 1.00 | **2.08** |
| CDR3524 |  | Putative aminotransferase | 1.00 | 1.00 | 1.00 | 1.00 | **0.44** |
| CDR3540 | *rnpA* | Ribonuclease P protein component (RNaseP protein) (RNase P protein) (Protein C5) | **2.08** | 1.00 | 1.00 | 1.00 | **1.93** |

Table B: qRT-PCR assays of genes differentially regulated during the kinetics of infection.

|  |  | **qRT-PCR** | | | |
| --- | --- | --- | --- | --- | --- |
| **Genes** | **Description** | 4 h | 6 h | 14 h | 38 h |
| CDR0043 | *thyA* | 1.63 | 1.41 | 0.78 | 1.46 |
| CDR0522 | *cotJB1* | 0.71 | 1.90 | 200.19 | 82.43 |
| CDR0582 | *tcdB* | 0.14 | 0.41 | 2.26 | 3.62 |
| CDR0583 | *tcdE* | 0.41 | 0.44 | 1.48 | 2.51 |
| CDR0584 | *tcdA* | 0.31 | 0.63 | 2.15 | 3.11 |
| CDR1740 | *ctn5* | 0.49 | 0.43 | 11.59 | 9.23 |
| CDR2214 | *gatC* | 8.76 | 10.18 | 2.65 | 7.12 |
| CDR2479 | *fbpA* | 0.88 | 0.89 | 0.91 | 0.55 |
| CDR2491 | *cdtA* | 0.27 | 0.40 | 0.99 | 0.90 |
| CDR2492 | *cdtB* | 0.21 | 0.31 | 0.89 | 0.64 |
| CDR2676 | *cwp84* | 0.80 | 1.28 | 2.03 | 0.78 |
| CDR2678 | *cwp66* | 0.50 | 1.12 | 1.06 | 0.59 |
| CDR3090 | *bclA2* | 0.68 | 0.92 | 2.68 | 3.67 |
| CDR3187 | *agr2* | 0.26 | 0.23 | 0.45 | 0.71 |

Expression value was compared to the time point 8h (p<0.05)

Table C: Regulation of colonization factor encoding genes during the kinetics of infection

Genes up-regulated by a fold change of 1.5 or more are in red and those down-regulated by a fold change of 1.5 or more are in green

| Gene ID CDR20291 (CD630) | Gene Name | Description Colonization factors | Fold change compared to 8h | | | | |
| --- | --- | --- | --- | --- | --- | --- | --- |
|  |  |  | 4h | 6h | 8h | 14h | 38h |
| CDR0440  (CD0514) | *cwpV* | Putative Hemagglutinin/adhesin | 1.00 | 1.00 | 1.00 | 1.00 | 1.00 |
| CDR0774  (CD0844) | *cwp25* | Putative cell wall-binding protein | **0.44** | 1.00 | 1.00 | 1.00 | 1.00 |
| CDR0802  (CD0873) |  | Lipoprotein adhesin | **0.41** | **0.45** | 1.00 | **3.80** | **0.27** |
| CDR2479  (CD2592) | *fbpA* | Fibronectin-binding protein A | 1.00 | 1.00 | 1.00 | 1.00 | 1.00 |
| CDR2670  (CD2782) | *cwp7* | Cell wall binding protein | 1.00 | 1.00 | 1.00 | 1.00 | 1.00 |
| CDR2676  (CD2787) | *cwp84* | Cell surface protein | 1.00 | 1.00 | 1.00 | 1.00 | 1.00 |
| CDR2678  (CD2789) | *cwp66* | Cell surface protein | 1.00 | 1.00 | 1.00 | 1.00 | 1.00 |
| CDR2680  (CD2791) | *cwp2* | Cell wall binding protein | 1.00 | 1.00 | 1.00 | 1.00 | 1.00 |
| CDR2681  (CD2792) | *secA2* | Protein translocase secA 2 | 1.00 | 1.00 | 1.00 | 1.00 | 1.00 |
| CDR2682  (CD2793) | *slpA* | Precursor of the S-layer proteins | 1.00 | 1.00 | 1.00 | 1.00 | 1.00 |
| CDR2683  (CD2794) | *cwp12* | Cell wall binding protein | 1.00 | 1.00 | 1.00 | 1.00 | 1.00 |
| CDR2685  (CD2796) | *cwp10* | Cell surface protein | 1.00 | 1.00 | 1.00 | 1.00 | **2.33** |
| CDR2686  (CD2797) |  | Putative fibronectin-binding protein | **2.33** | 1.00 | 1.00 | 1.00 | **2.27** |
| CDR2687  (CD2798) | *cwp9* | Cell wall binding protein | 1.00 | 1.00 | 1.00 | 1.00 | 1.00 |
| CDR2688  (CD2799) | *cwp8* | Cell wall binding protein | 1.00 | 1.00 | 1.00 | 1.00 | 1.00 |
| CDR3145  (CD3284) | *cbpA* | Collagen binding protein | 1.00 | 1.00 | 1.00 | 1.00 | 1.00 |

Table D: Carbohydrate transport and metabolism encoding genes regulated during the kinetics of infection.

Genes up-regulated by a fold change of 1.5 or more are in red and those down-regulated by a fold change of 1.5 or more are in green

| Gene ID CDR20291  (CD630) | Gene name | Description gene product | Fold change compared to 8h | | | | |
| --- | --- | --- | --- | --- | --- | --- | --- |
|  |  |  | 4h | 6h | 8h | 14h | 38h |
| CDR0134  (CD0135) |  | PTS system Lactose-cellobiose  IIA Component | 1.00 | 1.00 | 1.00 | 1.00 | 1.00 |
| CDR0135  (CD0136) |  | PTS system Lactose-cellobiose  IIB Component | **3.77** | 1.00 | 1.00 | 1.00 | **1.83** |
| CDR0136  (CD0137) |  | PTS system Lactose-cellobiose  IIC Component | 1.00 | 1.00 | 1.00 | 1.00 | 1.00 |
| CDR0137  (CD0138) |  | PTS system Lactose-cellobiose CHP | **4.44** | 1.00 | 1.00 | 1.00 | 1.00 |
| CDR0812  (CD0882) | *glgC* | Glucose-1-phosphate adenylyltransferase | **0.24** | 1.00 | 1.00 | 1.00 | **0.34** |
| CDR0813  (CD0883) | *glgD* | Glycogen biosynthesis protein | **0.30** | 1.00 | 1.00 | 1.00 | **0.39** |
| CDR0814  (CD0884) | *glgA* | Glycogen synthase | **0.32** | 1.00 | 1.00 | 1.00 | **0.42** |
| CDR0815  (CD0885) | *glgP* | Glycogen phosphorylase | **0.27** | 1.00 | 1.00 | 1.00 | **0.38** |
| CDR2207  (CD2318) |  | Putative phosphoglucomutase | 1.00 | **2.70** | 1.00 | 1.00 | **2.34** |
| CDR2209  (CD2320) | *rpiB1* | Ribose-5-phosphate isomerase1 | 1.00 | **2.14** | 1.00 | 1.00 | **2.05** |
| CDR2210  (CD2321) | *tkt4* | Transketolase | **2.20** | **2.50** | 1.00 | 1.00 | **3.61** |
| CDR2211  (CD23222) | *tkt* | Transketolase | **3.02** | **3.44** | 1.00 | 1.00 | **3.14** |
| CDR2212  (CD2323) |  | Putative carbohydrate dehydrogenase | **5.73** | **11.45** | 1.00 | **3.43** | **6.78** |
| CDR2213  (CD2324) | *gatD* | Putative galactitol-1-phosphate 5-dehydrogenase | **5.57** | **12.97** | 1.00 | **4.91** | **7.79** |
| CDR2214  (CD2325) | *gatC* | PTS system Galactitol IIC component | **8.57** | **9.70** | 1.00 | **7.68** | **6.74** |
| CDR2215  (CD2326) | *gatB* | PTS system Galactitol IIB component | **6.13** | **13.98** | 1.00 | **3.29** | **7.25** |
| CDR2216  (CD2327) | *gatA* | PTS system Galactitol IIA component | 1.00 | **6.31** | 1.00 | 1.00 | **3.71** |
| CDR2401  (CD2509) |  | 6-phospho-alpha-glucosidase | 1.00 | 1.00 | 1.00 | **2.59** | **6.07** |
| CDR2402  (CD2510) |  | PTS system Glucose-like subunit IIBC | 1.00 | 1.00 | 1.00 | **2.21** | **7.08** |
| CDR2403  (CD2511) |  | Transcriptional antiterminator | 1.00 | 1.00 | 1.00 | 1.00 | **4.89** |
| CDR2404  (CD2512) |  | PTS system Glucose-like subunit IIA | 1.00 | 1.00 | 1.00 | **2.54** | **7.40** |
| CDR2862  (CD3027) |  | PTS system Glucose-specific IIA component | 1.00 | 1.00 | 1.00 | **2.40** | **3.12** |
| CDR2865  (CD3030) |  | PTS system Glucose-specific IIBC component | 1.00 | 1.00 | 1.00 | **2.76** | 1.00 |
| CDR2866  (CD3031) |  | Transcriptional antiterminator | 1.00 | 1.00 | 1.00 | **2.04** | 1.00 |
| CD2913  (CD3074) | *gatY* | Tagatose-bisphosphate aldolase | **3.98** | 1.00 | 1.00 | 1.00 | 1.00 |
| CD2914  (CD3075) | *tagT* | PTS system transporter subunit IIABC | **4.28** | 1.00 | 1.00 | 1.00 | 1.00 |
| CD2913  (CD3076) | *tagK* | Tagatose (1)-phosphate-kinase | **3.85** | 1.00 | 1.00 | 1.00 | 1.00 |
| CDR2865  (CD3030) | *malX* | PTS system, maltose and glucose-specific IIBC component | 1.00 | 1.00 | 1.00 | **2.76** | 1.00 |
| CDR3136  (CD3275) |  | Phosphosugar isomerase | 0.37 | 1.00 | 1.00 | 1.00 | 3.51 |
| CDR3137  (CD3276) |  | PTS system mannose, fructose/sorbose IID Component | 1.00 | 1.00 | 1.00 | 1.00 | **2.43** |
| CDR3138  (CD3277) |  | PTS system mannose, fructose/sorbose IIC Component | **0.34** | 1.00 | 1.00 | 1.00 | **3.53** |
| CDR3139  (CD3278) |  | PTS system mannose, fructose/sorbose IIA Component | **0.40** | 1.00 | 1.00 | 1.00 | **4.04** |
| CDR3140  (CD3279) |  | PTS system mannose, fructose/sorbose IIB Component | 1.00 | 1.00 | 1.00 | 1.00 | **3.37** |
| CDR3263  (CD3441) |  | Aminotransferase | 1.00 | **3.62** | 1.00 | 1.00 | 1.00 |
| CDR3264  (CD3442) |  | Peptidase | 1.00 | **2.79** | 1.00 | 1.00 | 1.00 |
| CDR3265  (CD3443) |  | PTS system Lactose-cellobiose transporter subunit IIC | 1.00 | **2.18** | 1.00 | 1.00 | 1.00 |
| CDR3266  (CD3444) |  | PTS system Lactose-cellobiose transporter subunit IIB | 1.00 | **2.07** | 1.00 | 1.00 | 1.00 |
| CDR3267  (CD3445) |  | PTS system transporter Lactose-cellobiose subunit IIA | 1.00 | **3.25** | 1.00 | 1.00 | 1.00 |

Table E: Amino acid transport and metabolism encoding genes regulated during the kinetics of infection.

Genes up-regulated by a fold change of 1.5 or more are in red and those down-regulated by a fold change of 1.5 or more are in green**.**

| Gene ID CDR20291 (CD630) | Gene Name | Description | Fold change compared to 8h | | | | |
| --- | --- | --- | --- | --- | --- | --- | --- |
|  |  |  | 4h | 6h | 8h | 14h | 38h |
| CDR0830  (CD0900) | *opuCA* | Glycine betaine/carnitine/choline ABC transporter, ATP-binding protein | 1.00 | **2.14** | 1.00 | 1.00 | **0.43** |
| CDR0831  (CD0901) | *opuCC* | Glycine betaine/carnitine/choline ABC transporter, substrate-binding protein | 1.00 | **2.38** | 1.00 | 1.00 | **0.46** |
| CDR1501  (CD1603) |  | ABC transporter, permease protein | 1.00 | 1.00 | 1.00 | **2.93** | **2.73** |
| CDR1502  (CD1604) |  | ABC transporter, ATP-binding protein | 1.00 | 1.00 | 1.00 | **2.67** | **2.59** |
| CDR1503  (CD1605) |  | Hypothetical protein | 1.00 | 1.00 | 1.00 | **2.94** | **2.38** |
| CDR1824  (CD1904) |  | ABC transporter, permease protein | **0.30** | **0.31** | 1.00 | **7.17** | **4.59** |
| CDR2080  (CD2174) |  | Putative amino-acid ABC transporter, substrate-binding protein | 1.00 | 1.00 | 1.00 | **2.59** | **2.55** |
| CDR2081  (CD2175) |  | Putative amino-acid ABC transporter, permease protein | 1.00 | 1.00 | 1.00 | **2.32** | **2.44** |
| CDR2082  (CD2176) |  | Putative amino-acid ABC transporter, permease protein | **2.93** | 1.00 | 1.00 | **2.91** | **3.08** |
| CDR2083  (CD2177) |  | Putative amino-acid ABC transporter, substrate-binding protein | 1.00 | 1.00 | 1.00 | **2.40** | **2.38** |
| CDR2558  (CD2670) | *appF* | Oligopeptide ABC transporter, ATP-binding protein | 1.00 | 1.00 | 1.00 | **2.46** | **3.02** |
| CDR2559  (CD2671) | *appD* | Oligopeptide ABC transporter, ATP-binding protein | 1.00 | 1.00 | 1.00 | 1.00 | **2.51** |
| CDR2560  (CD2672) | *appA* | Oligopeptide ABC transporter, substrate-bonding protein | 1.00 | 1.00 | 1.00 | **3.34** | **5.00** |
| CDR2561  (CD2673) | *appB* | Oligopeptide ABC transporter, permease protein | **2.45** | 1.00 | 1.00 | **2.96** | **3.78** |
| CDR2562  (CD2674) | *appC* | Oligopeptide ABC transporter, permease protein | **1.92** | 1.00 | 1.00 | **2.19** | **2.45** |
